# Pea Breeding for Resistance to Rhizospheric Pathogens

**DOI:** 10.3390/plants11192664

**Published:** 2022-10-10

**Authors:** Osman Z. Wohor, Nicolas Rispail, Chris O. Ojiewo, Diego Rubiales

**Affiliations:** 1 Instituto de Agricultura Sostenible, CSIC, Avenida Menéndez Pidal s/n, 14004 Córdoba, Spain; 2 Savanna Agriculture Research Institute, CSIR, Nyankpala, Tamale Post TL52, Ghana; 3 International Maize and Wheat Improvement Center (CIMMYT), ICRAF House, United Nations Avenue—Gigiri, Nairobi P.O. Box 1041-00621, Kenya

**Keywords:** rhizosphere, soilborne disease, pea, breeding, fusarium, broomrape, rhizotrons, pathogens, resistance

## Abstract

Pea (*Pisum sativum* L.) is a grain legume widely cultivated in temperate climates. It is important in the race for food security owing to its multipurpose low-input requirement and environmental promoting traits. Pea is key in nitrogen fixation, biodiversity preservation, and nutritional functions as food and feed. Unfortunately, like most crops, pea production is constrained by several pests and diseases, of which rhizosphere disease dwellers are the most critical due to their long-term persistence in the soil and difficulty to manage. Understanding the rhizosphere environment can improve host plant root microbial association to increase yield stability and facilitate improved crop performance through breeding. Thus, the use of various germplasm and genomic resources combined with scientific collaborative efforts has contributed to improving pea resistance/cultivation against rhizospheric diseases. This improvement has been achieved through robust phenotyping, genotyping, agronomic practices, and resistance breeding. Nonetheless, resistance to rhizospheric diseases is still limited, while biological and chemical-based control strategies are unrealistic and unfavourable to the environment, respectively. Hence, there is a need to consistently scout for host plant resistance to resolve these bottlenecks. Herein, in view of these challenges, we reflect on pea breeding for resistance to diseases caused by rhizospheric pathogens, including fusarium wilt, root rots, nematode complex, and parasitic broomrape. Here, we will attempt to appraise and harmonise historical and contemporary knowledge that contributes to pea resistance breeding for soilborne disease management and discuss the way forward.

## 1. Introduction

Pea (*Pisum sativum* L.) is one of the oldest domesticated crops in the world. Pea is a self-pollinating diploid (2n = 14) with a haploid genome size of 4.5 Gbp. Pea served as a model crop in the hybridization work of Mendel, leading to the postulate on heritability [1]. Its centre of origin is primarily in the Near East with secondary diversification in the Mediterranean, Middle East and East Africa [2]. The taxonomy of the genus *Pisum* has been widely debated, but is generally accepted to contain three main species, *P. sativum*, *P. fulvum* Sibth and Sm. and *P.* *abyssinicum* A. Br. [1]. However, molecular evidence suggests that *P. sativum* subsp. *elatius* and *P. fulvum* are the two wild species from which domesticated forms are derived. The domesticated *P. sativum* and *P. abyssinicum* are considered derivatives of *P. sativum* subsp. *elatius* in two independent domestication events [3]. Therefore, pea has a very rich genetic diversity due to its typical broad wild progenitors.

This genetic diversity and germplasm reservoir are vital for pea breeding, thus a large collection is maintained in gene banks and is well-studied and preserved. These genetic materials include approximately 98,000 pea accessions distributed in about 25 gene banks, of which some 58,000 are unique accessions (https://www.genesys-pgr.org (accessed on 18 September 2022)). Despite the availability of these large collections, less than one per cent is made up of wild relatives [4]. Yet, wild species are a valuable reservoir of resistance traits, particularly useful for pre-breeding and disease resistance breeding. Hence, it is critical to properly characterize and preserve them to maximise their utilization [5,6]. Pea wild relatives have already been successfully explored and used in pea breeding [7,8,9]. So, resistance breeding can be accelerated by the introgression of desirable wild alleles, complemented with the adoption of novel techniques and tools for pea precision breeding [10,11].

The cultivated forms of pea are grouped into green pea for human consumption, dry pea and fodder pea for animal feed [12]. Pea is the fourth most important grain legume worldwide, following soybean, peanut and dry bean. The current annual production estimates for the year 2020 are around 14.6 million metric tons (MT) of dry pea and 19.8 million MT of green pea with yield averages of 2.0 t/ha and 7.9 t/ha, respectively, projected to increase in the coming years. The leading producers are Canada, the Russian Federation, China, India, Ukraine, the United States of America, Australia, Ethiopia and Tanzania [13]. The current crop productivity must be increased to feed the continual population growth envisaged to reach 10 billion by 2050, despite the expected reduction in arable land. It is consequently crucial to explore smart agriculture and suitable land use to ensure climate change mitigation and food security [14,15]. Accordingly, pea is a legume crop candidate in this race for food security owing to its multipurpose low input requirement, nitrogen-use efficiency, soil economy amendment and biodiversity attributes. Moreover, it has a nutritional purpose with valuable sources of dietary fibre, high proteins (25%), mineral nutrients and many health benefits [16]. Likewise, the mutualistic association of pea with the N_2_-fixing soilborne rhizobium reduces chemical fertilizer inputs, attaining yields with minimal impact on the ecosystem [17,18]. Pea and related legumes as pre-crops in a rotation programme can also provide important benefits for the environment by liberating nitrogen for the succeeding crops, and can serve as a buffer to cereal crop farmers in terms of price instability and crop failure [19,20,21].

Despite these benefits, the pea crop can be constrained by various diseases that severely affect yield and seed quality [4,12]. The most challenging disease limitations are the rhizospheric diseases found in the vicinity of host plant roots within the bulk soil, where they incubate and infect their hosts [22]. Yet, the available management techniques are limited—those available are either not economical or unhealthy to the environment, and most research efforts against pea rhizospheric diseases only present an incomplete resistance. Therefore, this review aims to consolidate the progress from various research findings on rhizospheric pathogens and serve as a resource for sustainable pea soilborne disease management and future advancements in pea resistance breeding. We delve into the most important pea rhizospheric diseases, including fusarium wilt (*Fusarium oxysporum* f. sp. *pisi*), root rot complex (fusarium, aphanomyces, thielaviopsis root rot, seed-based rhizoctonia and pythium rots), parasitic broomrapes (*Orobanche crenata*), and nematodes complex (cyst, root-knot and root lesion nematodes). Here, soilborne diseases and rhizospheric diseases are synonymously used to refer to diseases found incubating and surviving in the soil niche—with emphasis on those diseases that are closely associated with or infecting the host pea root zone (rhizosphere).

## 2. Host Pea-Rhizosphere-Microbial Interactions and Stress Amelioration

**Figure 1:**
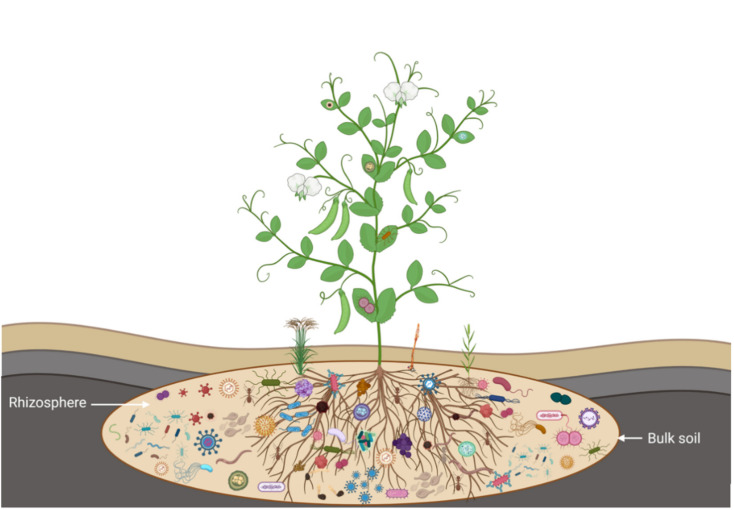
Rhizosphere–microbiota configuration in pea roots showcasing a multitude of interactions. Represented here are mutual associations such as rhizobia bacteria and host pea nodules—fixing nitrogen for pea growth while pea maintains bacteria nourishment, and beneficial associations such as earthworms soil burrow activities—improving soil aeration and fertility for pea, and antagonistic associations such as parasitic broomrapes and parasitic nematodes affect pea production. (Illustration made in ©BioRender—biorender.com).

The rhizosphere harbours a great diversity of microorganisms involved in plant–microbe and plant–rhizosphere–microbe interactions. These microorganisms include plant pathogenic, beneficial, antagonistic, and synergistic associations [23]. The selective release of exudates from host plants activates and sustains specific rhizobacterial communities at the locality of the host’s rhizosphere [24]. For instance, the successive rotational cultivation of pea and other pulses with cereals modified soil structure and increased the diversity of the rhizosphere microbial community [25], suggesting that legumes pose a much stronger influence on the selection of their rhizosphere than cereals. Accordingly, pea plants influence the configuration of microbial populations in the rhizosphere systems (Figure 1).

Advanced high-throughput sequencing tools can identify host plant–rhizosphere–microbial associations to provide clarity regarding the resistance mechanisms in legume root diseases [26]. This high-throughput information has been used to map rhizosphere related traits in pea. For instance, 16S rRNA gene amplicon sequencing and quantitative polymerase chain reaction (qPCR) techniques were used to analyse a pea microbial community—the abundance of proteobacteria, rhizobacteria spp., and discovered plant-growth-promoting genes indicated that pea plants shape their rhizosphere for nutrient uptake and stress amelioration [27]. Pea nodules were also profiled using both responsive genetic constructs and hormones, suggesting that enhanced cytokinin during rhizobium symbiosis is associated with bacterial penetration and the subsequent differentiation of bacteroid within plant cells [28]. Hence, plant growth regulators could enhance microbial activity. Moreover, a study established that a supernumerary chromosome of *Nectria haematococca* carries pea-pathogenicity-related genes and a trait for pea rhizosphere competitiveness. These genes can enable nutrient absorption and stress tolerance in pea [29]. The role of rhizosphere traits in delineating toxic compounds has also been elucidated, postulating that the soil–microbial–plant continuum can mitigate aluminium toxicity through the solubilization of soil phosphates, thereby increasing nutrient availability in the pea rhizosphere [30]. A study of pre-penetration mechanisms of *Fop* revealed that pea root’s secretion of the toxin phytoalexin-Pisatin into the rhizosphere could reduce pathogen pressure [31].

In tomatoes, rhizosphere traits were used to identify beneficial bacterial genes involved in the metabolism of plant polysaccharides, iron, sulphur, trehalose, and vitamins, whose genetic variation was linked to specific quantitative trait loci (QTLs) [32]. The information on these beneficial genes can be exploited to facilitate the mapping of pea rhizosphere traits. Furthermore, plant-growth-enabling microbes that form an active part of organic matter or biofertilizers can enhance crop yield and complement plant resilience against pathogens [33,34]. The notable mutual association between pea root nodules and the bacteria *Rhizobium leguminosarium* for N_2_ fixation and the release of root exudates, which promote the infection of parasitic broomrapes, is well-established [35]. A recent study suggested that prolonged organic soil amendment can harness phosphate-solubilizing microbes in the rhizosphere. These beneficial bacterial endophytes within the pea rhizosphere can improve insoluble nutrient uptake [36]. The rhizosphere deposition of host-root exudates disseminates complex extra-cellular DNA molecules, and antimicrobial proteins to neutralise pathogenic threats and avoid host tissue intrusion [37]. However, some dispositions of secreted effectors by rhizosphere pathogens may inhibit plant defences by affecting RNA helicase involved in root defence and development [38]. An understanding of this linkage and the increasing evidence of genetic variation controlling pea–microbial interactions can be exploited for pea breeding. Current insights into the genetic basis of host pea–rhizosphere interplay are key for defence against disease complexes, providing opportunities for pea resistance breeding [39]. Therefore, the resistance against rhizospheric diseases can be achieved by the indirect selection of rhizosphere associated traits. These can be used as pointers and their correlated attributes incorporated into breeding programmes [40,41]. Hence, a holistic understanding of the entire soil ecosystem is needed for resistance breeding strategies to unravel the obscure and complex defence mechanisms against rhizospheric diseases [26,42].

## 3. Pea Rhizospheric Diseases

Novel plant diseases will continue to emerge due to changing climate conditions, farming systems and feeding modes of pathogens and parasites. As a result, previously non-economic pathogens may become important in range and severity [43,44]. The outbreak of any disease in the rhizosphere depends not only on the pathogen, but also on its interaction with the host and the environment, in the so-called pathogen–host–environment disease triangle [45]. Control strategies must be formulated to disrupt this triangle disease balance, either by altering the environment, reducing pathogen pressure or increasing host resistance [46]. Unfortunately, rhizospheric pathogens are more difficult to manage than their aboveground counterparts since they have the ability to survive on plant debris and soil in modified forms [26]. These pathogens can be harnessed and characterised with modern tools to estimate their historical emergence, spread and evolution in the rhizosphere in order to improve diagnostics and the design of efficient control measures [47,48].

The production of grain legumes such as pea is severely affected by soil-infecting pathogens. Pea rhizospheric or soilborne diseases can cause yield losses of between 80 and 100% when not controlled [49,50]. The resemblance of soilborne disease symptoms to other biotic factors further complicates the diagnosis strategy. Thus, the use of chemical, biocontrol and cultural means of control is crucial, although insufficient in most instances. Hence, it is necessary to find the most efficient, economic and environmentally friendly approaches using resistant cultivars [51]. However, breeding for resistance to rhizosphere diseases is complex, as the quantitative nature of resistance is often partial in nature and difficult to select. On the other hand, monogenic resistance is easy to select and has been achieved in some soilborne diseases of legumes, but the rapid evolution of pathogens can easily break down the existing levels of resistance. So, the understanding and evaluation of soilborne diseases by efficient identification and screening techniques is a requirement for the implementation of effective control strategies and resistance breeding [49,52]. Therefore, in this review, some of the important pea rhizospheric diseases are deliberated.

### 3.1. Fusarium Wilt 

The genus *Fusarium* encompasses many, mostly soilborne, species reported to affect animals, humans and plants alike, including endophytes, saprobes and pathogens [53]. The genus was initially classified into 70 species on the basis of morphology, biology and phylogenetic criteria [54]. A more recent evaluation reduced their number to nine species, of which *F. oxysporum* is the most important, broadly affecting many crops [39]. Within *F. oxysporum*, the forma specialis *pisi* (*Fop*) primarily infects pea [55,56] and grass pea [57]. Similar to the other f. sp. of *F. oxysporum*, *Fop* has the ability to colonise and infect the soil, organic material residues, host roots and shoots leading to vascular wilt and yield penalties [57,58]. Four races have been described for *Fop* so far, of which races 5 and 6 are mostly found within the Americas, while races 1 and 2 are found worldwide [59,60]. Races 3 and 4 were initially defined as distinctive variants, prior to their reclassification as aggressive variants of near wilt *Fop* race 2 [61]. This explains why there are four races. Generally, symptoms of the disease are typically progressive from older to younger leaves/stems resulting in stunting, yellowing, necrosis to wilting, and finally, plant death [55,62,63]. *Fop* can be disseminated over long distances through the transport of contaminated soil samples by animals, farm technicians, machinery or infected crop seed lots [64,65]. Fungi show survival plasticity in most soil conditions, through the formation of thick-walled chlamydospores that can hibernate in the soil for years. This persistence is further enhanced through aggressive saprophytic mode in host plant debris and via released microconidia and macroconidia, which aid new infections and dispersal [60,66]. Therefore, a clear understanding of the disease cycle and mode of spread is required for the efficient implementation of diagnostic and control strategies.

**Figure 2:**
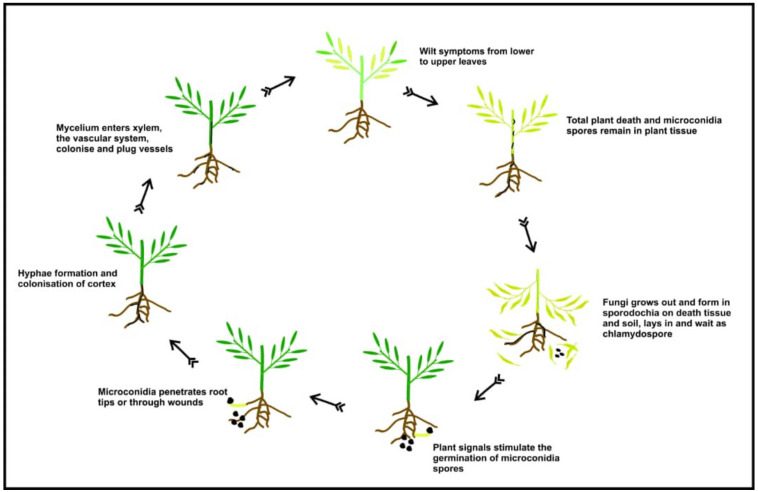
A pictorial presentation of fusarium wilt life cycle.

The disease cycle (Figure 2) initiates in the soil, where the spores, in the form of chlamydospores, lie in wait for unsuspecting hosts. Pea roots emit unknown signals that trigger spore germination in the presence of conducive microclimatic factors. After germination, germlings grow towards pea roots by chemotropism. Once in contact with the host roots, the elongating spore attaches to the root and then enters through root tips, root hairs and wounds. The invading hypha then directly penetrates the root epidermis without the formation of a differentiated penetrative structure. The penetrating hypha then advances through the root cortex until it reaches the vascular tissues and enters the xylem vessels through the pith. Upon reaching the vascular stele, the fungus modifies into an endo-phytic mode of colonization using the xylem vessels to colonize the upper part of the host plant. At this stage, the fungus remains exclusively within the xylem vessels, taking nourishment from the host. The extensive growth of the fungus in the xylem leads to the interruption of the water flux, causing the distinctive wilt symptom manifestation on leaves and ultimate death of the host. Upon plant death, it begins to grow out of the xylem vessel to reach the surface of the dead tissues, where it produces chlamydospores, which disperses into the soil, causing looming infestation in subsequent seasons [55,57,58].

Once the disease cycle and dynamics are well-understood and proper screening methods are identified, managing the disease becomes simple. Fortunately, the fungi can easily be recovered from infected soil and plant tissues and maintained on an axenic medium, such as potato dextrose broth/agar, for its morphological and molecular characterisation. While this has aided the identification and diagnosis of the pathogen, its control strategies continue to be a huge challenge [67,68]. The disease can be managed by enforcing policy compliance via host plant material quarantine at border points of entry from hotspot zones to contain the pathogen [69]. A number of agronomic and cultural practices, such as crop rotation, fallow cropping and fertilization, can improve soil integrity to suppress *Fop* by reducing the pathogen inoculum [57]. For instance, micro-fertilizers inhibit the production of the mycotoxin, fusaric acid, which is a key pathogenic element in *Fop* infection [70,71].

Biological control through the use of natural enemies and suppressive organisms can be used to control *Fop*. The use of bioagents such as *Trichoderma* spp. and *Bacillus*-based antimicrobials have been reported as potent mediators for managing *Fop*, and also stimulates systemic host plant resistance and development [72,73]. Further, chemical control such as soil fumigation with ammonium bicarbonate or plant treatment chemicals can be effective for *Fop* control [74]. In the absence of resistant cultivars, the integrated application of these control methods would be more effective on *Fop.*

The identification of resistance sources within germplasms and a combination of practical phenotypic evaluation standards and host resistance genotyping can improve sustainable *Fop* control strategies in the long term [57,75,76]. So, the knowledge of the fungal patho-systems, genetics and physiological variations can inform sustainable resistance breeding strategies [52,64]. Qualitative resistance for *Fop* race 1, 5 and 6 have been identified and deployed into pea varieties using classical breeding [62,77]. Resistance to race 1 is located in the pea linkage group (LG) III in the vicinity of a SCAR marker Y15_999_*Fw*, which could be useful for improving selection for race 1-resistant cultivars through marker-assisted selection (MAS) [78,79]. Resistance to *Fop* race 2, which causes late symptoms, regarded as “near wilt” in the field, appears to be quantitative in nature in both pea and grass pea [55,68,80]. Accordingly, the resistance identified so far against race 2 is more complex, with two minor loci identified in LG III (*Fnw3.1* and *Fnw3.2*) and a major allele at LG IV (*Fnw4.1*) providing a basis for MAS in pea breeding against this race of *Fop* [81]. Pea association mapping for agronomic and quality traits revealed 71 significant marker associations linked to 25 valuable traits, including disease resistance [82]. In addition, a recent genome-wide association study (GWAS) in grass pea identified 17 single-nucleotide polymorphisms (SNP) associated with *Fop* resistance, seven of which were assigned to pea chromosomes 1, 6, and 7 by sequence homology hits [57,83]. This knowledge can improve the use of molecular strategies for *Fop* resistance breeding. Further studies showed that, host resistance is a result of the development of physical and chemical barriers within pea root tissues, leading to cell wall and xylem reinforcement to block pathogen growth [60]. Furthermore, these studies found that some pea accessions harboured a constitutive pre-penetration resistance inhibiting *Fop* germination, which might contribute to reduce pathogenic pressure [31]. These mechanisms can be exploited in pea breeding against *Fop*. Although monogenic resistance is easier to handle by breeding, there is the risk of breakdown by the constant mutation of the pathogen that already led to the emergence of the distinct races of *Fop* [49,79]. Thus, it is essential to continuously search for novel sources of resistance to complement and reinforce the resistance of elite cultivars, hence the need to pay attention to durable resistance [55,68].

### 3.2. The Rots Complex

Root, seed and seedling rots are a group of soilborne pathogens that affect pea production in many areas. Pea root rots are mainly caused by *Aphanomyces euteiches*, *Fusarium solani*, and *Thielaviopsis* spp., whereas seed and seedling rots are caused by *Rhizoctonia* spp. and *Pythium* spp. [63]. Some viruses and bacteria are also cited to provoke root rots [34]. In this paper, they are together referred to as the rots complex. These rot complexes can be instigated by a single pathogen or a cocktail of pathogens causing seed/seedling decay, root and foot decay, and necrotic wilts [84]. Consequently, the rots complex leads to severely stunted host growth, loss of vigour and yield injury to crops [85]. They also stimulate pre-emergence and post-emergence damping-off and other root rot pestilences [50]. Commonly used management practices, such as soil treatments, adjustment of planting time, seed vigour and quality, seed treatment with fungicides and crop rotation have not been broadly successful [86]. Therefore, the deployment of integrated techniques in the rhizosphere with host-plant-associated microbes could promote the control of the rots complex [87].

#### 3.2.1. Aphanomyces Root Rot

The soilborne oomycete *Aphanomyces euteiches* is a highly specialised legume pathogen causing root rots, which result in economic yield losses. This pathogen is considered an important disease in pea-growing economies and affects the plant at all developmental stages [88]. Infection is initiated by zoospores and oospores stimulated to germinate by pea root exudates; they penetrate pea roots and colonise host tissues, forming a network of mycelia [89]. The mycelia take assimilates from the host plant, causing root damage and a yield penalty of up to 86% in heavily infested pea fields [90]. These effects are further aggravated through the action of other pathogenic enzymes when found in associations with root rots [49]. The root-rot-afflicted plants become dwarfed and water-stressed due to the progressive development of watery lesions and dark brown roots with cortex decay, resulting in subsequent yellowing and wilting of the upper part of the host plant [91]. After the host roots and tissues decay, spores are re-injected into the soil and some remain on the plant material debris, all serving as an inoculum awaiting another infection cycle. These spores are so hardy that they can remain in the soil for years in the absence of the host. The survival of the pathogen is further prolonged by secondary and volunteer weeds serving as alternative hosts that also increase the inoculum bank in the soil [63,90,92]. In addition to their longevity, the pathogenic spores can be disseminated over long distances by running water and other farm operations [87].

The resistance to *A. euteiches* in pea is of quantitative nature with few cultivars containing good levels of partial resistance, and there are no efficient control methods [93,94]. However, several applications of biocontrol products with antagonistic soil bacterial strains such as *Bacillus* spp., *Pseudomonas fluorescens*, *Pantoea agglomerans*, and *Lysobacter capsici* were found to suppress aphanomyces root rot in pea [95]. Although biological control agents have better efficacy under control conditions than under field trials, the integration of a mixed inoculant of different strains could be more effective than a sole application, improving their large-scale efficacy [96]. For instance, the combined application of *Lumbricus terrestris* and *Bacillus velezensis* was found to reduce *A. euteiches* infection in pea, with the response being attributed to soil disturbance and direct antagonistic feeding [97]. The efficacy of some biological agents is comparable to chemical applications. In turn, the application of the endophyte fungus *Clonostachys rosea* was found to reduce pathogenic aphanomyces intensity by 76%, similar to fungicide treatments [84]. Although certain chemicals can be effective, their cost and environmental concerns make them less preferred. The fungicides metalaxyl-M and fosetyl-AL were reported to increase seedling emergence and delay the infection of *A. euteiches* in field pea [84,90], while ethaboxam fungicides were found to suppress *A. euteiches* of pea in North America [98]. On the other hand, cultural methods of soil testing can help to avoid infested areas, and the use of *Brassicaceae* and *Poaceae* families in rotation management practices can minimize the spread of *A. euteiches* in pea fields [89,90].

Advanced breeding techniques for resistance against *A. euteiches* are promising. For example, molecular marker technology was used to identify and release eight F8-derived recombinant inbred lines (RIL) of pea with improved partial resistance to *A. euteiches* and acceptable agronomic attributes [99]. QTL association studies revealed partial resistance genes against *A. euteiches* in pea, reporting a reliable QTL (*Aph1*) in LG IVb, which explains 47% of the variation as a potential option for pea improvement [100]. In a meta-analysis of partial resistance genes to *A. euteiches* in four main sources of resistance in pea, seven highly consistent genomic regions with potential for MAS were identified, and candidate genes underlying six meta-QTL regions were found in collinearity between pea and *Medicago truncatula* genomes [101]. QTL validation was also performed to confirm *A. euteiches* resistance in different pea backgrounds in a backcross to generate near-isogenic line (NIL) populations. These allowed for the development of several breeding lines carrying distinct levels of resistance by marker-assisted backcrossing [102]. Similar association studies also identified 11 markers significantly associated with *A. euteiches* resistance, confirming and refining the location of previously identified QTLs, and uncovering four novel resistance QTLs [103,104,105]. In addition, one significant SNP was mapped to the major QTL *Ae-Ps7.6*, associated with both *A. euteiches* resistance and pea root system architecture traits [104]. Recently, another stable and major QTL was mapped to an approximately 20.0 cM region on pea chromosome 4, which was identified as the most consistent region conferring partial resistance to *A. euteiches* [106]. These efforts together with some transcriptomic pathway studies that identified expressed candidate genes at the cellular level [92], guided the improvements of *A. euteiches* resistance towards precision and marker-assisted breeding.

#### 3.2.2. Fusarium Root Rot

Fusarium root rot has a wide host range, attacking the host at the cotyledon and tap root zones within ground clearance down to the rhizosphere. In pea, it can be caused by *Fusarium solani* f. sp. *pisi* (*Fsp*) and *Fusarium avenaceum* complex [61]. These fungi are distinct from *Fop*, though they sometimes combine to form a complex and associate with other diseases to infect pea [63,107,108]. Although *Fsp* was initially described as the main causal agent of fusarium root rot in pea, *F. avenaceum* seems to be gradually gaining more prominence over *Fsp*, as *F. avenaceum* was found to constitute 45- 48% of recovered isolates from analysed infected samples of pulse crop residues [109]. The infecting fungal chlamydospores can lay dormant in the soil for many years until their germination is activated by exudates from imbibed host seeds and nourishment obtained from the germinating seedling. Fusarium rot fungi can also freely form cocoon-like colonies in the soil rhizosphere, which increase the pathogen fitness and longevity in the soil [85]. The infection of the pathogen is through the stomata of epi-hypocotyl zones and downstream into the root system. Pathogen severity and spread are further exacerbated by conducive soil moisture, low soil fertility gradient and other pathogenic stresses [65,85,110]. Fusarium root-rot-infected plants display root lesions, vascular tissue decolourisation and root dysfunctions similar to other root rot complexes [39,111].

Control management involves the cultural use of extensive rotation regimes to minimise the inoculum bank, use of good agronomic practices to improve soil fertility and root growth, the avoidance of soil compaction, and the use of good quality seeds [56,110,112]. Some biocontrol agents, such as rhizosphere mycoflora and *Bacillus* spp., have been used as potential solutions to suppress *Fsp* [73,113]. Interestingly, *Pseudomonas* spp. and *Bacillus* spp. can produce volatiles with an antibiotic effect on *Fsp*, whilst *Pythium oligandrum*, *Trichoderma* spp. and *Streptomyces* spp. exhibit hyper parasitism and mycoparasitism against the fusarium root rot [114]. Currently, there are no effective chemical fungicides for the control of *Fsp* [110].

Resistance to *Fsp* has been reported as a quantitative genetic trait with some partial resistance obtained in pea germplasms. The accessions with pigmented flowers were observed to have a tendency of greater partial resistance to *Fsp* than white-flowered cultivars [115]. A pea RIL population was used to further identify one major QTL and five minor QTLs for *Fsp* resistance and one QTL against *F. avenaceum.* The major QTL, *Fsp-Ps*_2.1_, was located within a 1.2 cm interval on chromosome 6 and explained 44–53% of the total variance, while the five minor QTLs were more loosely located on chromosome 1, 4, 5 and 7 [65]. Similarly, four QTLs associated with resistance to *F. avenaceum* in a pea RL population were identified by QTL analysis. These markers identified a key QTL on chromosome 7 that explained 21.7% of the variance in resistance [116]. Another study identified a QTL associated with resistance to *Fsp*, where the QTL was found flanking markers *AA416* and *AB60* on LG VII with 39% explained variance [117]. A more recent study using SNP-derived markers from differentially expressed genes and two RIL populations detected additional QTLs on chromosomes 2 and 3 and confirmed two minor QTLs on chromosome 5 [34]. The application of these identified loci is expected to improve pea resistance breeding. In addition, an extensive RNASeq approach comparing eight tolerant and susceptible pea accessions to *Fsp* identified more than 42,000 differentially expressed genes (DEG) in response to *Fsp* [118]. These DEGs could complement pea breeding efforts for resistance to fusarium root rot.

#### 3.2.3. Black Root Rot

The causal pathogen of black root rot, *Thielaviopsis basicola*, has a wide host range affecting various crops. On a global scale, black root rot is of minor importance [119] but can gain prominence over time with increased inoculum when not controlled. *T. basicola* forms a complex with other soil microbes to exacerbate injury to host roots. Host infection is initiated by pathogenic spores in soil and plant debris, which attack host seeds and root surfaces to spread and cause harm to plants. The spores infect root hairs, and the germ tube penetrates the root cells. The hyphae differentiate into feeding structures (haustoria-like) to absorb nutrients from host cells and disrupt water flux, causing cortical cell death [63]. Field infection is aided and disseminated by farm tools, water flow downstream and aggravated by high soil moisture with high temperatures. *T. basicola* causes pea roots to turn dark-brown or to develop dark lesions and necrosis at the root base, leading to water deficit, stunting growth and plant dysfunction [63,108]. 

A recent phylogenetic analysis showed that *T. basicola* includes cryptic sister species, and it was proposed to rename them as *Berkeleyomyces basicola* and *B. rouxiae* [120]. This suggests that the pathogen could be evolving, and highlights that a more detailed understanding is needed to establish control strategies. The main management strategy is to avoid the spread of the pathogen into non-infected areas. Furthermore, crop rotations and biocontrol measures can limit the pathogen build up [63]. Although little is understood about the management of the pathogen in the pea crop, lessons can be learned from other crops. In bean fields, cover cropping and green manure are reported to reduce root rot intensity [121]. Compost and other organic amendments are also useful in controlling root rots [108]. Chemicals have not been feasible against this disease, hence breeding for resistance is deemed economically appropriate but it is not considered a major concern in pea [63]. However, there are concerns about the emergence of new variant of *T. basicola* that may become a major threat to pea in the future due to changing climate conditions. *T. basicola* resistance is controlled by a single dominant gene in tobacco [122], and similar resistance genes (R-genes) were found to reduce root lesions in tree cotton (*Gossypium arboreum*). Three resistance QTLs, which together explained a total phenotypic variation of 32.7%, have been identified in *G. hirsutum.* A subsequent synteny analysis of these significant QTL regions with Arabidopsis revealed a total of 624 genes, including 22 pathogen defence genes and 36 stress-related genes that could correspond with *T. basicola* resistant QTLs [123]. These associated genes and novel disease discovery mechanisms are valuable for understanding the resistance of *T. basicola* in crops including legumes. Although the mechanism of resistance for *T. basicola* in pea is not sufficiently studied, it is anticipated that a similar mode of quantitative resistance may be present in pea as observed in other crop species.

#### 3.2.4. Rhizoctonia Root Rot

Rhizoctonia root rot is caused by *Rhizoctonia* spp. which are sporeless and largely consist of hyphae, hyphal propagules and sclerotia. They are classified under filamentous hymenomycetes with an asexual mode of reproduction comprising a host of unrelated species. To distinguish them, *Rhizoctonia* spp. isolates are ascribed to distinct anastomosis groups (AG) via a somatic incompatibility test. The most important group is composed of isolates of *Rhizoctonia solani* [124]. Most AG groups of *R. solani* can cause root rots in pea, but the AG4 is the most frequent and most virulent [63]. The economic merit of this pathogen spans most pea-growing areas, and it is harmful to a broad host range of plants. In severe cases, the effects of the pathogen induce yield losses from about 75% to total crop failure [125]. The germination of *R. solani* sclerotia is triggered by host roots or imbibed seed exudates. The elongating hyphae then enter the seedling by means of soft spots or wounds. The appressorium attaches to host cells and supports pathogen growth, where the pathogen secretion further weakens the host cells to enable colony growth and host root tissue invasion. This eventually causes cell death and the production of sclerotia mass as inoculum for the next pathogenic cycle [61,63,125]. *R. solani* causes soggy lesions on juvenile shoots and roots, adventitious shoots formation, as well as the damping-off and dieback of roots and seedlings. This is exacerbated by high-moisture regimes, poor soil and drainage conditions [63]. *R. solani* is mostly responsible for poor germination and growth due to seed/seedling infections in the rhizosphere. The effects of the disease are visible in the whole plant, and pathogen coexistence with other complexes further complicates diagnosis [126,127]. *R. solani* is reported to be the prevalent isolate causing severe root rots in pea in the Colombia and Washington basins of the USA. The recovery of the pathogen from plant samples and molecular identification by laboratory techniques are key to the pathogen diagnosis [125,128]. *R. solani* can live for lengthy periods in the soil and plant materials as infectious hyphae or hibernate in saprophytic mode [124]. The pathogen exhibits opportunism to non-host species and weeds, further expanding its host scope in a diverse manner through secondary hosts [63].

Complete control strategies of *R. solani* are lacking, hence it is more practical to foster preventive and resistance strategies [125]. Consequently, it is paramount to use eco-friendly techniques to manage the pathogen. Thus, several measures for the sustainable improvement of pea against this rot complex are well-established [52]. Resistance to seedling rot caused by *R. solani* is linked with seedling epicotyl thickness and age, with younger seedlings being more susceptible to infection [63,125]. Thus, good husbandry practices with the use of certified disease-free seeds, seed treatment with fungicides or starter macro-nutrients are useful control strategies. This would improve seedling vigour and limit the inoculum of *R. solani* [129].

In a long rotation and no-till programme, a low population of *R. solani* was observed in legumes compared to cereal cultures, suggesting that long rotations prior to planting can reduce the pathogen activity in pea [130]. The most significant biocontrol study undertaken on pea is the combination of *Rhizobium leguminosarum* and *Trichoderma* spp. isolates to manage *R. solani* [127]. These beneficial mycoparasitism processes mostly involve the synthesis of cell wall lytic enzymes. Promising studies on other crops include the use of bio-formulation of rhizobacteria mixtures as seed treatment significantly suppressed *R. solani* in sunflower [131], *Trichoderma* species were effectively used for *R. solani* management in chickpea [132], and the *Bacillus* spp.-mediated synthesis of selenium nano-particles was also found to be efficient to attenuate *R. solani* in faba bean [133]. Furthermore, *R. solani* fungal genome mycovirus sequence breakthroughs have advanced control methods [134,135]. These fungal genome sequences have already steered the identification of a new effective candidate genus (Betapartitivirus) to help understand the patho-dynamics and enhance control strategies [136]. These mycoviruses have the ability to reduce the mycelial growth of many fungi [135]. Some microbial markers associated with pea roots have also been elucidated and show promise as a *R. solani* control [129]. Thus, they could be useful as biocontrol agents to control *R. solani* in pea. Chemical controls are mostly limited but the use of bavistin 50 WP, and provax-200 fungicides show a complete inhibition of *R. solani* colony growth in pea [137]. Fumigants with the active ingredients of thiophthalimide (captan), quinone outside inhibitors (azoxystrobin), succinate dehydrogenase inhibitors (fluxapyroxad), demethylation inhibitors (ipconazole), aromatic hydrocarbons (tolclofos-methyl) and phenylpyrroles (fludioxonil) have been used as seed treatments to manage *Rhizoctonia* spp. These compounds are mostly single-site inhibitors, so it is recommended to combine them with other treatments to reduce the risk of pathogen resistance [63]. The integration of management techniques could be more efficient against *R. solani* since host plant resistance is limited [26]. It has been demonstrated that canola lines expressing pea defence gene *DRR206* confer resistance to *R. solani*. These constitutively mediated defence genes are often effective against different pathogen species and can be exploited for *R. solani* resistance in pea [138]. In a study of *R. solani* AG2-2IIIB strain, it was shown that lysin motif (LysM) effector protein contributes to virulence through the evasion of chitin-triggered immunity, and the obstruction of this protein has the potential to protect pea against *R. solani* [139]. In the model legume, *M. truncatula*, ethylene-mediated signalling and the overexpression of isoflavonoid proteins have been identified to improve resistance against *R. solani.* These expressed defence genes can protect legumes against root pathogens [140]. The understanding of the role of these disease response proteins can be used to complement pea resistance breeding against *R. solani*.

#### 3.2.5. Pythium Seed/Seedling Rot

This root rot is caused by the oomycetes *Pythium* spp., including *P. ultimum* and *P. aphanidermatun*, which broadly affect pea. The disease is severe under conducive moist and poor soil conditions, causing poor germination, bare patches, and a watery-brown discoloured root system [84,141]. The pathogen produces oospores that reside in the soil and on plant tissues, infecting pea at the pre-emergence and post-emergence stages to cause seed/seedling rot and damping-off [63]. The host plant attracts the zoospores, which encyst on the root surface. The cyst–spore germinates and the mycelia invade and propagate within the root tissue, taking up nutrients to produce sporangia and more oospores to aid their survival [142]. The produced propagules can be spread by animals and farm operation tools and can remain in the soil for several infection cycles [50,130]. Their survival structures exist as saprophytes and persevere in the soil as colonizers of organic matter. Therefore, a thorough understanding of the disease pathway will be essential to establish successful control strategies. This can be elucidated using analyses of the genetic relatedness among isolates from diseased plants, water and soil samples [142].

Control of this pythium complex is difficult due to the interwoven nature of many species. Damping-off can be controlled using integrated disease management strategies involving cultural, biocontrol and host-resistance breeding [39,143,144]. For cultural management practices, it is important to avoid poorly drained soils, maintain good soil health, good tillage, and use disease-free seeds [133,144]. Biological control using rhizobium strains has been shown to offer protection against pythium damping-off in pea [84]. Other biological agents of *Bacillus* spp., *Trichoderma* spp., *Pseudomonas* spp., and *Streptomyces* spp., are appropriate beneficial species that are useful against pythium rots at small scale, but they may not be viable for larger areas [63]. Apart from biocontrol agents, compost has been reported to be effective against pythium rots in vegetables and legumes. Soils with enforced organic matter content also suppress the disease manifestation [112,144]. Chemical treatment with appropriate fumigants, including mefenoxam and ethaboxam, can be effective against pea [63] if long-term effects are discounted.

In pea, resistance to *Pythium* spp. is not readily available. Pea cultivars with large seeds have been reported to give vigorous seedling stands, exhibiting better resistance than those with small seeds [145]. Consequently, using quality seeds with maximum vigour is crucial for healthy seedling emergence to reduce the risk of infection [63,146]. In soybean, partial resistance has been achieved through the identification of two QTLs against *P. ultimum*, explaining 13% and 16% of the phenotypic variance, respectively [147], and two other QTLs (0.8534 and 0.6955 heritability) against *P. aphanidermatun* [148]. An additional GWAS on soybean lines was used to identify 7 SNP markers and 5 QTLs (9.7–16% of variance) associated with partial resistance to *Pythium* spp [149]. Similarly, in snap bean, polygenic resistance was identified in a major QTL associated with *P. ultimum* resistance, which explained 25-49% of variability [150]. Although it is difficult to identify race-specific resistance genes against *Pythium* spp., quantitative gene expression approaches have proven valuable for developing pythium complex resistant varieties [151]. The motif Arg-Xaa-Leu-Arg (RxLR), encoding protein effectors in *Pythium* spp. that exhibited a suppressive activity, was identified and shown to protect host plants by promoting cell death [152]. This knowledge is useful for developing potential candidates for pre-breeding efforts in pea genetic resistance.

### 3.3. The Nematode Complex

Soil nematodes are micro-worms with unsegmented bodies, less than 2 mm in diameter. The genus comprises up to 20 genera, some of which are associated with the rhizosphere of field pea roots and related legumes [61,63]. They have a wide host range, infesting and causing injury to many plant species. The most economically damaging nematodes on pea are the *Heterodera goettingiana* (cyst), *Meloidogyne* spp. (root knot) and *Pratylenchus* spp. (root lesion) [153,154], making up the pea nematode complex under consideration.

The infection cycle of nematodes is initiated by free-living eggs and juveniles in the rhizosphere of an ideal host or volunteer host plants. Eggs hatch and juveniles detect and penetrate host roots, guided by host plant signals. They then feed on the root tissues and grow to form a colony, which causes the most damage. The infection cycle is similar for most nematodes, except the pea cyst, where the mode of feeding and colony structure of females undergo shedding. Pea cyst nematode eggs also requires a host stimulus to hatch, and they are very mobile. Contrary to cysts, root knot nematodes do not depend on host stimuli to hatch and are largely spot feeders, whilst females of root lesion nematodes do not shed at root penetration and are highly migratory. In general, female nematodes are the most destructive and the producers of eggs for their sustained survival advantage. Thus resistance techniques should be targeted at limiting female populations since males are absent in most species [153], except in lesion nematodes, which assume a sexual mode of reproduction. The nematode complex can continue their unabated infection spree on available hosts and thrive for many years [61,63,155,156,157].

Nematode-affected plants show signs of dwarfism, patches of necrosis and yellowing, loss of vigour approaching those of soil health deficiencies and a reduction in host root system function [158]. The affected areas also differ in intensity by soil fertility gradients, soil type, soil gravimetric contents, host plant resilience and the parasitic-ecosystem dynamics [155]. In pea, parasitic nematodes generally interfere with rhizobium activity, which reduces nitrogen fixation and leads to yield penalty. In addition, they can serve as vectors of other diseases, exposing plants to further stresses [63]. Nematodes are detected through keen field observations, soil sampling and analysis to estimate the economic threshold per gram of soil. The minimum threshold of 3 to 15 eggs per gram of soil is enough to engulf an entire pea field, resulting in serious yield penalties of up to 50% if not controlled [63].

Control strategies for these soilborne parasitic nematode complexes are limited, thus there is a need to prevent the movement of the pathogen into non-infected areas by adhering to field phytosanitary and cultural protocols. Given the difficulty to completely control nematode complexes, the overall goal of the applied control strategies is usually to prevent parasitic nematode populations from reaching economic injury levels [159]. Cultural methods of tillage and crop management have been recommended to reduce nematode incidence [130,159,160]. Some fungi are nematophagous and they prey on nematode eggs as food, which can be exploited for biocontrol management [161]. Mycorrhizal fungi have also been reported to inhibit nematode entry by aiding hormonal balance in the host to improve nutrient uptake, and different strategies of biocontrol have been reported [161,162]. For chemical control, fumigation with dichloropropene-based fungicides and non-volatile nematicides can provide a good control of these nematodes to improve pea yields [163]. Host resistance efforts, using infested fields and controlled pot experiments show promise [157,164,165,166]. However, for decades, the interest in plant parasitic nematodes has mainly focused on biological control and host–parasite interactions [154].

#### 3.3.1. Pea Cyst Nematodes

Cyst nematodes represented by isolates of *Heterodera goettingiana* is an important economic parasite of pea, which can survive for over 10 years without a viable host. Cyst nematode populations are widespread in Europe, Russia and the Mediterranean belt [63]. Besides peas, many legume species are suitable hosts of this nematode, including faba bean, vetch and black medick [167]. Infection is promoted by non-aerated soil, a suitable moist climate and host plant vulnerability. In addition to depriving host plants of nutrients and water, they also reduce the plant’s natural defences and synergise with other harmful microbes to injure plants [161]. Host exudates trigger pathogen activity and root penetration, where juveniles feed on root cells and moult. Upon its entry into the plant roots, the nematode intracellularly migrates to the vascular bundle, where it selects a cell to become its initial feeding site, and subsequently develops into a syncytium [168]. Cysts are often found within feeding sites, but they can be transported by running water, farm equipment and other soil fauna, which increase the infection and spread of the pathogen [63].

The best management strategies are to use fallow systems, host resistance and trap crops to activate nematode activity leading to suicidal natural death and predation [45]. This makes it important to study nematode morphological diversity, population dynamics and ecology to promote sustainable management strategies [169]. Late planting may reduce the infection rate of the pathogen due to reduced moisture conditions towards the end of the season [166]. It was reported [63] that the utilization of longer rotation systems in the absence of host plants for up to 6 years can drastically reduce cyst nematode populations. However, for the success of rotations, a better understanding of the host range of nematodes is required to prevent incubation in the absence of the main host plant [167]. The incorporation of 10% aldicarb nematicides into infected soil before planting has been reported to control *H. goettingiana* in pea [170]. The nematicide oxamyl also improved pea growth against *H. goettingiana* [171]. Pea-recommended herbicides such as prometryne are reported to hinder egg-hatching ability and affect female *H. goettingiana* development [172]. This could protect plants against nematodes in the absence of resistant cultivars.

The screening of a pea collection under controlled conditions led to the identification of potential sources of resistance. The results show that five *Pisum abyssinicum* accessions, a *P. arvense* accession, and a *P. elatius* accession demonstrated a moderate resistance to *H. goettingiana* [173]. Subsequently, a histological observation of some pea accessions showed that resistance to *H. goettingiana* was caused by the rapid degradation of cysts through a hypersensitive response and associated cell death. The resistance reaction was also connected with lignification and suberisation processes surrounding the necrosed area of infection [174]. Reverse transcriptomic expression analysis revealed that the polygalacturonase-inhibiting protein improves pea defence against *H. goettingiana*. The gene *Pspgip1* was expressed in infected cortical cells and localised in cells bordering cyst-induced syncytia in resistant pea roots, confirming that this gene was key in preventing *H. goettingiana* establishment [175]. Further molecular studies suggested that the involvement of lipoxygenase and the polygalacturonase-inhibiting protein in cyst nematode induced resistance in pea [174,176]. Although these findings improved the understanding of the cyst nematode resistance reaction, they have not been effectively exploited to create resistant elite cultivars through breeding [63,177].

#### 3.3.2. Root Knot Nematodes

**Figure 3:**
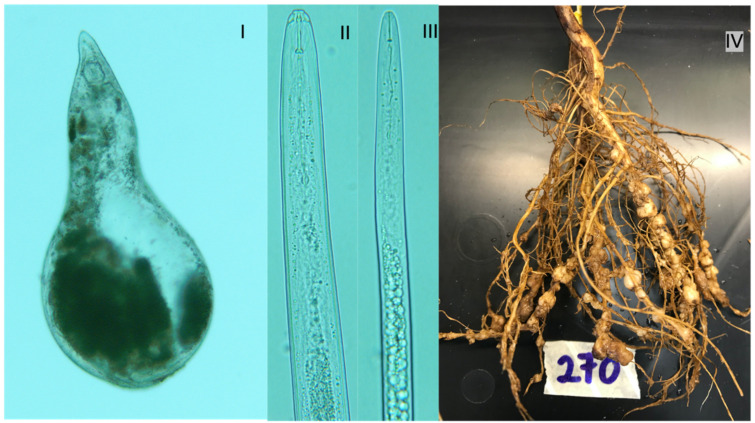
Microscopic view of (**I**) female *M. incognita*, (**II**) male *M. incognita*, (**III**) juvenile *M. incognita* (source P. Castillo, IAS-CSIC) collected from (**IV**) galled root symptoms on soybean plant (O.Z. Wohor IAS-CSIC).

Root knot disease is caused by *Meloidogyne* spp. These nematodes have a wide host range, being capable of infesting most crop species, including legumes. Amongst the *Meloidogyne* spp. complex, the *M. incognita* (Figure 3) is nature’s most flourishing plant pathogen. Infected plants become malnourished, stressed with a loss of vigour, and could prematurely shed inflorescence [153,155]. *Meloidogyne* spp. are obligate endoparasites and stationary feeders but can be spread by farm activities and running water [45]. They possess a feeding conduit, which operates as a molecular sieve for ingesting assimilates. The accumulation of these assimilated compounds forms the characteristic giant cell chambers (nodule-like) on host roots. Their effect reduces host crop physiological functions through root system deprivation of essential nutrients.

These giant cells manifesting in all *Meloidogyne* spp. indicate that nematode–host-plant-associated genetic factors are involved in the formation process [153], thus enabling the exchange of biochemical compounds between the pathogen and host plant. The *Meloidogyne* spp. infection complex in association with *F. oxysporum* increases fusarium wilt severity and other biotic stresses [178]. Therefore, it is essential to understand the genetics and dynamics of the host–parasite associations to identify management approaches.

The management of root knot nematodes requires integrated strategies, including cultural methods of prolonged fallowing [157], avoiding host plant monocultures and abstaining from areas with secondary host weeds such as alfalfa [63,167]. The beneficial associations of some microbes can be exploited to enhance the availability of nutrients and useful minerals to improve plants’ health and immunity to induce resistance to root knot pathogens [114,179]. Some of these microbial isolates from rice rhizosphere are reported as biocontrol agents against root knot nematodes [180]. Panth et al. [114] reported findings of important biocontrol agents such as *Gliocladium catenulatum*, which produce toxins effective against nematodes, *Purpureocillium lilacirium* as parasites on nematodes, and some beneficial *Mycorrhizae* spp. that compete with nematodes for space and resources. In chemical control, low-risk organic chemicals from essential oils of *Ocimum basilicum* at 125 mg/L were effective against *M. incognita*, resulting in a 70% mortality rate in a bioassay test conducted under controlled conditions [181]. The use of chemical fumigants such as 1,3-dichloropropene alone or in combination with methyl isothiocyanate has great potential for the management of root knot nematodes in pea [163].

Host resistance to root knot nematodes is considered a viable long-term goal, although little resistance has been found and released for cultivation so far [63]. A number of criteria have been used to screen for resistance, such as the rating of infected roots on a 1–5 scale, gall severity index [166], or a 0–4 index scale and the reproduction index, calculated as the ratio of total nematode population count per host on the initial inoculated population count [182]. The use of reproductive index led to the identification of pea accessions with a moderate-to-high level of partial resistance to *M. incognita* and *M. javanica* [63,182,183,184]. The advanced gene manipulation techniques of using nematode protein effectors contributed to elucidate resistance signalling pathways against *M. incognita*. These pathways can modify the host cell wall and regulate stress signalling and hypersensitive response [185]. In *Arabidopsis thaliana*, it was revealed that the actin-depolymerizing factor (ADF) is upregulated in the giant feeding cells during host infection and the knockdown of a specific ADF isotype inhibits *Meloidogyne* spp. proliferation. Hence, limiting the expression of this gene in host plants can prevent the development of *Meloidogyne* spp. [186]. The draft genome of *M. incognita* provided insights into some parasitic adaptive genes and their effects on immune-competent hosts [187]. These may be applicable through gene expression studies to identify *Meloidogyne* spp. resistance sources to promote pea breeding.

#### 3.3.3. Root Lesion Nematodes

Root lesion is elicited by *Pratylenchus* spp. The most prolific and common root lesion nematodes affecting pea are *P. neglectus* and *P. thornei*. Root lesion nematodes are difficult to manage due to the high invasion of both juveniles and adults at point of entry and inside root tissue [63]. The juvenile stage 4 (J4) can assume a dormant non-feeding form in a self-regulatory mode under unsuitable conditions, while waiting for the appropriate conditions to rejuvenate. All the larval stages are active feeders on root cells, and they reproduce in a sexual mode. Quantitatively, one pathogen per gram of soil is capable of rapidly ravaging pea fields [188], validating their aggressiveness and spread in the rhizosphere.

Their persistence is further strengthened by their parthenogenic nature since females can produce eggs in the absence of males [189]. They are migratory and can navigate between feeding sites for nourishment and spread exudates and pathogens between host plants. These exudates and movements to and from feeding sites cause characteristic lesion symptoms in roots. Unlike root knot nematodes, they do not exhibit the usual root bulging signs. Instead, they degenerate the root epidermal cells causing serious root damage and yield losses [190,191].

Early detection is key for the prevention and management of this nematode. So, there is the need to frequently examine pea fields to avoid new infections and establish standard disease management strategies to prevent the spread of lesion pathogens [45,166]. Although crop rotation can be efficient for controlling lesion nematodes, it is less effective against root lesions due to their persistence and ability to infest both cereals and legumes [63,130,192]. Sterilization methods using gamma irradiation (7.5 k Gy), aerated steam (80 °C) and methyl bromide fumigation successfully eliminated *P. thornei* in vertisols in Australia [193]. The non-volatile nematicides (aldicarb, oxamyl, carbofuran, thionazin and fenamiphos) provide good control against these nematodes [163,194]; however, these chemical sterilizations may not be economically feasible in large pea fields [63]. Therefore, the combination of different disease management techniques should be applied to efficiently reduce root lesions severity. 

Disrupting the ideal environments of the pathogen and searching for host resistance is needed to control *Pratylenchus* spp. [63,165]. Despite limited available information on resistance against *Pratylenchus* spp., some progress has been made. For instance, a screening for low pathogenic growth and reproduction index reported moderate levels of resistance in pea accessions against *P. nanus* [188]. Resistance can also be determined from soil samples by comparing the initial pathogen population density over the end of season population density. This is more representative than using lesion symptoms [166]. Furthermore, moderate levels of resistance against *P. thornei* and *P. neglectus* were found in chickpea cultivars and wild relatives [189,195]. The broader characterisation of the resistance mechanisms acting in these legumes could have a positive impact on pea breeding against root-lesion nematodes.

### 3.4. Root Parasitic Weeds: Broomrapes

**Figure 4:**
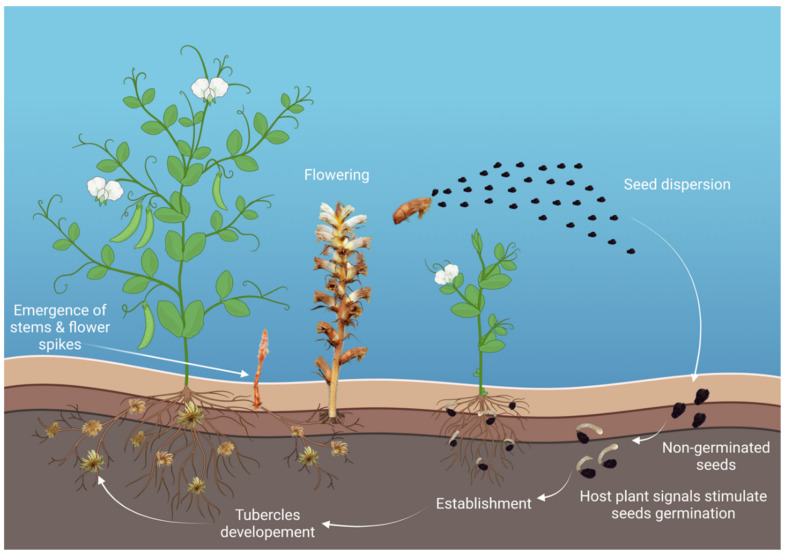
Broomrape infection life cycle, exhibiting below- and above-ground scenarios. (Illustration made in ©BioRender—biorender.com).

Broomrapes are soilborne root parasitic plants that constrain crop production. They are widely distributed in the temperate regions of the Mediterranean and Middle East [196]. The family *Orobancheae* consists of about 150 species, some of which can broadly affect important crops, with the species *Orobanche cumana* and *O. crenata* being specific to a few plant genera, whilst *Phelipanche aegyptiaca* and *P*. *ramosa* are broadly found in nature. The legume-damaging species are *O. crenata*, which is distributed widely; *P. aegyptiaca*, which is restricted to the Eastern Mediterranean and the Middle East; and *O. foetida*, so far limited to Tunisia and Morocco [196,197]. Between them, *O. crenata* is the most important root parasitic weed constraining pea production since it is capable of causing up to 100% yield losses if not controlled [198]. *O. crenata* is a holoparasite without chlorophyll and therefore feeds solely on host plants for all of its nourishment. Germination is usually initiated by seed preconditioning under conducive environments triggered by strigolactones and other chemical signals typically produced by host roots [199]. Upon germination, the emerging radicle grows towards the plant roots by chemotropism. This is guided by the concentration gradients of the host stimuli. The germination radicle then attaches to the root and penetrates the host root vascular system for nourishment and development. This leads to the formation of specific nodules and tubercles on the root surface from which the broomrape shoots are differentiated and emerge from the soil to flower, allowing seed formation and dispersal to continue its endless lifecycle (Figure 4) [200,201,202,203]. Broomrape seeds can remain dormant in the soil rhizosphere for many years in the absence of a host.

A series of broomrape management strategies has been proposed, including field sanitation and containment, prevention/avoidance of spread, and agronomic and chemical treatments to reduce seed banks in the soil [197,203,204]. Cultural control measures have shown promise against *O. crenata* through crop rotations/trap cropping or intercropping with allelopathic crops and field sanitation. Intercropping pea with oat, fenugreek, or berseem clover has been shown to reduce *O. crenata* infection [205,206]. Control techniques that trigger suicidal seed germination have long been suggested but have not reached a commercial stage due to difficulties in the formulation and delivery of germination stimulants or trap crop use [202].

Chemical control of broomrape by foliar applications of glyphosate at low rates is recommended for faba bean and vetches, but pea is highly sensitive to glyphosate-based herbicides [200]. However, pea has better tolerance to pre-emergence and post-emergence imidazolinone herbicide treatments, although no complete control is provided, and treatments are mostly less effective on earlier sowing dates [207]. The interspecific association between the host pea and parasite makes the selective use of herbicides practically impossible. Moreover, the efficacy of herbicides requires repeated applications that are often not cost-efficient for farmers, given the low-input cultivation system of pea [196].

Biological control has also been proposed to protect pea against broomrapes. Some rhizospheric-associated beneficial microorganisms such as arbuscular mycorrhizal fungi, *Azospirillum* spp., *Azotobacter* spp., *Bacillus* spp., *Pseudomonas* spp. and other rhizobacteria can provide protection to pea by suppressing *O. crenata* germlings [208]. These rhizobacteria can reduce the parasitism of *O. crenata* on pea roots via induced chemical and mechanical blockages in the host xylem [209]. Likewise, some arbuscular mycorrhizal fungi (AMF) can reduce the seed germination rate of *O. crenata* [35], and *O. cumana* seed establishment [210]. The use of non-pathogenic *F. oxysporum* strains has also been reported to control *O. cumana* and *P. aegyptiaca* [85]. Despite these positive results at the laboratory scale, biological controls are yet to be used in large-scale commercial applications. Since there is no single efficient control mechanism for managing *O. crenata*, the most promising mechanism is integrated pathogen management with different control strategies and breeding for resistant cultivars [204].

Breeding approaches for resistance to *O. crenata* mostly target host resistance and herbicide resistance [211]. Broomrape resistance breeding is challenging due to the low levels of resistance detected in existing pea germplasms and the low heritability of the resistance [200,212]. Therefore, highly variable germplasms and an understanding of the parasitic host specificity and population dynamics are needed for implementing efficient breeding strategies. Fortunately, unlike reported biotypes in *O cumana* [210], there is no clear proof of the existence of biotypes for *O. crenata* species thus far [213]. Therefore, the effectiveness of *O. crenata* resistance found in a host genotype may not diverge from the environment in which it is grown.

The genetic base for resistance to broomrapes is suggested to involve a complex inheritance governed by minor genes with small effects [214,215]. Resistance was confirmed to be largely quantitatively inherited and steered the identification of partial resistance in wild pea and landraces [200]. The achieved resistance has since been successfully bred into pea cultivars [9,216]. This highlights the importance of wild relatives as sources of useful alleles in pea breeding. As an alternative to a host resistance control strategy, *O. crenata* resistance may be achieved by breeding for early maturity lines, which have the advantage of escape to outcompete the parasite [200,217].

The current use of applied breeding is leveraging biotechnological tools to develop efficient markers to help breeders speed up cultivar release to farmers [4,211]. Molecular markers were utilised in an F_2-3_ population to detect two QTLs (*Ocp1* and *Ocp2*) for broomrape resistance, which together explained 20% of phenotypic variance [214]. A subsequent study on a RIL of the same biparental population elucidated four useful QTLs associated with field traits and broomrape resistance mechanisms assessed in vitro. The QTLs individually explained about 10 to 17% and 8 to 37% of the phenotypic variance for field and in vitro attributes, respectively [215]. These molecular markers provide the basis for linked trait association for marker-assisted selection (MAS). However, further saturation of the detected loci is often required to bridge the gap between alleles and the genomic regions flanking markers to enable marker-assisted breeding (MAB) [197,218]. The use of microarrays, transcriptomics and proteomics provided quality gene expression indicators to elucidate *O. crenata* resistance. For example, a gene expression profiling of *M. truncatula* against *O. crenata* using a transcriptome pathway found many functional genes and validated 35 associated defence genes acting at the early penetration and late tubercle necrosis stages [218]. A proteomics analysis using gel electrophoresis and mass spectrometry untangles 49 differential gene spots of defence- and stress-related proteins against broomrape infection in model *M. truncatula* [219]. This confirmed previous proteomic studies that reported 22 expressed gene spots related to defence response against *O. crenata* in pea [220]. Therefore, the recent advances in omics and molecular breeding have the potential to change the paradigm of pea breeding [4] and elucidate the molecular basis of *O. crenata* resistance.

## 4. Breeding Enabling Approaches for Disease Resistance

Progress has been made in identifying sources of resistance to pea rhizosphere root diseases. The effects of these diseases are determined by multiple soil microbial factors and host plant attributes [129]. Conventional breeding approaches are expected to remain viable in disease screening for many decades and beyond. However, these traditional methods need to be complemented with novel genomic and biotechnological approaches for better results. The breeding strategy for pea is similar to other self-pollinated crops. These can be implemented by germplasm assembly and the selection of ideal parents for breeding new cultivars. The development of improved cultivars for resistance against a single pathogen is often a simple process. However, this requires a good source of resistance with efficient and extensive screening approaches to provide sufficient selection pressure [201,221].

### 4.1. Phenotyping

Phenotyping entails the morphological description of visually developing plant parts, physiology, stress resistance and agronomic parameters linked to yield. Phenotyping is crucial to plant breeding since it is the main basis for selecting lines for developing new cultivars [14]. Novel genomic sequencing technologies have become more affordable, resulting in quality reference genomes and huge genomic datasets, yet phenotyping remains a limiting factor in accessing these gains [222,223]. Disease resistance screening is performed by subjecting breeding lines to uniform disease pressure. This enables the discrimination of contrasting lines into resistant, susceptible and intermediate classes. The selection of desired traits requires an ideal environment to permit full phenotypic expression of resistant alleles [224]. Traditional plant phenotyping based on visual observations and manual data capture is predisposed to evaluation errors [225]. So, breeders are required to utilize the understanding of genotype and environment relations to improve phenotypic accuracy and reduce these errors [226]. Currently, efficient and simple specialized systems are beginning to accelerate pea resistance breeding under optimal growth conditions [227].

#### 4.1.1. Field and Controlled Condition Phenotyping

Under field and controlled conditions, detailed descriptions of screening methods for legume root diseases [39,49,145], parasitic weeds [228], nematodes [177], and their application in pea breeding have been reported. Field screening enables the simultaneous evaluation of genetic materials at a large scale under natural environmental conditions, although it allows less control of the pathogen and the environmental factors. Under controlled conditions, disease resistance assessments are more accurate, and environmental factors, such as light, water, nutrients and temperature, are better controlled than under field conditions [229]. Here, seedlings, in vitro cultures, detached plant parts and young/short cycle plants are mostly preferred. However, there is often a poor correlation between the results obtained in the greenhouse or laboratory and those collected under field conditions due to field plasticity, and the high genotype by environment interaction under field conditions [44,49]. Thus, refinements in screening methods are continuously being implemented to improve accuracy. For instance, Bani et al. [55] modified the root-dip method of inoculating soilborne fungal pathogens on pea roots and incorporated a two-way rating scale of disease severity at the whole-plant and leaf scales. This method improved fusarium wilt disease examination and provided a comprehensive description of pea resistance to *Fop*. Similarly, refinement of the pot and in vitro screenings revealed many potential pea lines with some levels of resistance against broomrapes based on avoidance, low host induction, suicide/necrosis of germlings, and exudate/germination inhibition mechanisms [8,230,231]. Recently, a non-inversive greenhouse system was shown to be useful for screening field pea against aphanomyces root rots to elucidate biogenic markers for the pathogen control [232], thus providing an advanced and efficient phenotyping regime for pea disease assessment. 

#### 4.1.2. High-Throughput Phenotyping

Nowadays, low-throughput screening is considered a bottleneck to phenotyping and often requires specialised breeders’ expertise, thus high-throughput techniques are beginning to replace visual screening [224,233]. High-throughput phenotyping gives the opportunity to overcome visual assessment bias and difficulty to access plant traits. This would improve selection intensity and allow cost-efficient precision screening of large numbers of samples [234]. Currently, intelligent above- and below-ground vehicles are equipped with remote sensors to efficiently capture quantitative and geographic data across broad areas to improve breeding programmes [235]. The modern use of smart cameras, unmanned aerial vehicles (UAV), near-infrared reflectance spectroscopy (NIRS)/infrared systems, X-ray tomography, artificial intelligence (AI), machine learning (ML) data [236,237], and other specialised precision intelligent robotic systems are starting to deliver reliable phenotyping [238]. The advent of 5G technology could further accelerate rapid data capture, data transfer and interoperability between intelligent platforms, which would aid real-time cloud data storage. This is expected to eliminate unreliable data storage and multiple data transfer misrepresentations—transposition/inversion and substitution of figures manually inputted between systems [239]. The application of high-throughput imagery phenotyping has elucidated early pea vigour traits and improved pea breeding [240]. Likewise, novel infrared imaging technique has been exploited to evaluate differential pea and *M. truncatula* lines against their respective *F. oxysporum* pathogens to discriminate between resistant and susceptible plants. This could be useful for obtaining first-hand disease information before the development of disease symptoms in host plants [67,241]. Consequently, high-throughput phenotyping tools have overcome previous time-consuming constraints. These innovative tools coupled with industrial-scale genotyping could be used to mine germplasms for important traits. This can improve scientists’ use of genomics, bioinformatics and biotechnological methods [223,242,243].

#### 4.1.3. Innovative Rhizotrons for Rhizosphere Phenotyping

**Figure 5:**
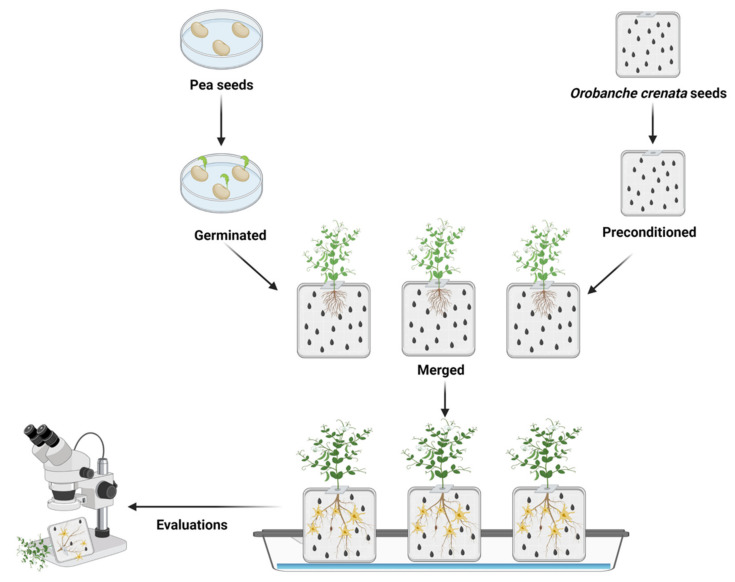
A mini-rhizotron set up for *O. crenata* screening, indicating pre-germination of host plant seeds and preconditioning of parasitic seeds to merger for association and evaluation. (Illustration made in ©BioRender—biorender.com).

In the past, conventional phenotypic techniques to assess root disease, including soil excavation and profiling soil sections served their purpose, albeit being disruptive to plants [244] and presenting a high heterogeneity and interference ambiguity [245]. To circumvent these difficulties, researchers have, over the years, developed several innovative systems to achieve the in vitro characterisation of root development and root responses to stresses [246]. These innovative tools specifically designed for root examinations are broadly termed as rhizotrons or mini-rhizotrons (Figure 5). They are basically a plant roots observatory system for continuous monitoring and non-destructive sampling of the rhizosphere functions at different developmental stages of crops [247]. Rhizotron observatories can be useful for improving pea-breeding programmes. However, light capture bias in most enclosed systems may hinder comparative analysis with field data [248]. So, rhizotrons have to be accurate to mimic soil conditions to enable results to be representative of field scenarios [246]. Rhizotrons come in various configurations with assorted materials, forms, shapes and sizes, comprising basic wooden boxes, soil trenches with plastic insertions, glass walls to recent opaque imaging tools, and other underground observatory facilities [249,250].

Recently, breakthroughs in the non-disruptive examination of plant roots have been made by innovative high-throughput phenotyping methods and simple root analytic tools [247]. Topical innovative soil-filled rhizoboxes have been established for rhizospheric exploration. These have been used to determine root physicochemical properties based on hyperspectral imaging [251]. Likewise, an innovative minirhizotron was presented as ‘SoilCam’ for automated root system monitoring and root imagery analysis to promote crop performance [252]. Rhizotrons can be integrated into speed-breeding protocols [14,253,254] to improve pea resistance breeding against rhizosphere pathogens. An example of cheap rhizotrons for practical pea root examinations are simple Petri dishes filled with soil media sandwiched between fiberglass paper and the host roots, which have been adopted for determining the resistance response against broomrapes in pea [8,212,217] and other legumes [255,256]. Similar minirhizotrons are effective for evaluating resistance against parasitic *Striga hermonthica* in cereals [257,258,259], and assessing the biocontrol of broomrapes using rhizobium strains [208,209,210]. Therefore, rhizotrons have great implications for pea improvements towards the control of *O. crenata* and may be useful for the study of rhizobacterium dynamics, parasitic nematodes, and other microfauna interactions with pea roots.

### 4.2. Trait Discovery and Pre-Breeding for Resistance Breeding

Gene discovery and pre-breeding are crucial for the continuity of pea breeding programmes. The success of breeding programmes is dependent on broader genetic pools with a high genetic diversity and environmental adaptability. Germplasms from gene banks are a great source of genetic diversity, which is key for crop improvement [11,196,223]. This variability can be sourced from wild relatives, landraces, breeding lines, and mutants, which are very useful for pre-breeding, and can stimulate breeding. Understanding the genetics of cross-compatibility among germplasm can reduce breeding barriers to enable the full utilisation of pea genetic diversity in breeding. All *Pisum* sp. are readily crossable to the cultigen, *P. sativum* ssp. *sativum*. Therefore, the use of pea wild relatives with appropriate breeding regimes can generate multitudes of pre-bred genotypes, which can further be utilised in mainstream breeding for continuous accelerated gains [6]. Although selection in breeding is labour-intensive, breeders need to evaluate their decisions with scientific accuracy and economic merit to improve genetic gain [260,261].

### 4.3. Genotyping: Genomic Tools and Genetic Breeding Approaches

Genomic approaches using high-throughput genomic information in the areas of genome sequencing, data resequencing, genome-wide markers, genetic maps, QTLs, diagnostic markers, and omics strategies (transcriptomics, proteomics, metabolomics biomarkers), assist with and direct multiple breeding strategies [4,233,262]. The genetic revolution provided by the next-generation sequencing (NGS) platforms ensures the development of approaches, such as genotyping by sequence (GBS), diversity array technology sequencing (DArTseq), ribonucleic acid sequencing (RNA-Seq), whole-genome sequencing (WGS), among others, which have improved the quality of marker technologies [263,264]. This led to the discovery of extensive single-nucleotide polymorphic (SNPs) markers [265] with a huge potential for pea improvements [233]. These novel platforms have already guided the identification of heritable QTLs contributing to phenotypic variance in pea resistance breeding [102,266,267,268]. These high-throughput techniques also enabled the quantitative elucidation of nematode population composition [269], and pea genetic diversity studies [270,271].

The upscaling of QTL mapping techniques is useful but mostly confined to detecting gene variants in bi-parental segregants. Therefore, GWAS based on the non-random linkage of loci in haplotypes enhances high-resolution mapping of quantitative traits. Thus, improving and complementing traditional bi-parental mapping and validation of QTL alleles to facilitate MAS [223,272]. The efficiency of GWAS is determined by LD, population structure and genetic diversity, and has the power to uncover causative genes missed in QTL mapping populations [104,273]. Thus, GWAS has successfully aided the identification of novel variant–trait associations for breeding valuable disease resistance traits in pea and other legumes [82,83,104,274,275]. Marker–trait association analysis has been used to uncover significant linkage among pea inbred lines for resistance to *Fop*-R1. Five candidate genes were identified, and three of those markers (*Fw_Trap_480*, *Fw_Trap_340*, *Fw_Trap_220*) were tightly linked to the *Fw* locus at 1.2 cM, thus offering the potential for MAS in pea [276]. The associated genetics with nanopore sequencing (SQK-RAD004) have been used to manipulate genomic regions controlling pea pod colour and other traits, which are expected to accelerate the pan-genome variation in pea Mendelian traits [277]. An association study was also used to elucidate the linkage between aphanomyce-resistant genes and late flowering, and flower colour variants in pea [103]. Novel QTL approaches can be applied to reduce the limitations of GWAS since a marker may not be in LD with the causal genes in succeeding generations [278]. This can be achieved by targeting haplotypes to accelerate trait introgression and QTL stacking via nested association mapping (NAM) with multiple association studies (QTL+GWAS). The use of advanced–backcross loci (AB-QTL) for allele delivery and expressed loci (eQTL) analysis can help explain the variation of SNPs in gene expression and pan-genomics for dissecting entire sets of gene families [263]. These advanced QTL techniques can enhance trait associations to narrow the genetic distance between markers and alleles for MAB.

The advances in proteomic pathways revealed gene expressions associated with disease resistance in pea. This has improved the understanding of the genetic basis for broomrapes and *Fop* infection studies [220,279,280]. Recently, genomic selection (GS) has become valuable in predicting the breeding values of cultivars without phenotypic information, using genomic data obtained from the prediction models of a training population. This has been successfully explored in pea predictive breeding [281] and selection for pea yield and abiotic stress tolerance [264,282]. Genomic selection can be used to identify multiple traits to improve pea rhizosphere disease-resistance mechanisms through genomic-assisted breeding [283]. Hence, these genetic tools and procedures improve the capacity of breeders for the uptake of biotechnology and reduce the gap between genomics, molecular and conventional breeding strategies [245,284]. For instance, the post-genome reverse genetics technique of gene silencing (RNAi) and ‘targeted induced local lesions IN genomes’ (TILLING) for gene deletion and point mutation can confirm gene function to accelerate a selection of desirable traits [4,285]. This has facilitated the characterisation of mutant nodulation traits in pea [286]. The construction of ethylmethan sulfonate reference mutant populations and data bases using phenotypic and sequence data have improved the availability of pea TILLING genes. These mutants can be scanned with the BLAST tool to locate similar gene families for different useful traits [287]. These mutation libraries are reported to improve virus-induced gene silencing (VIGS) implementation in pea [288]. VIGS of a yeast protein *MtSTP13* has been found to repress pathogenesis-related gene expression and enhance powdery mildew susceptibility in *M. truncatula*, while its transient overexpression improves the resistance of pea against powdery mildew [289].

The novel revolutionary gene editing CRISPR/Cas9 and single-guided RNA sequence (sgRNA) are powerful optimised tools for gene functional studies in many crops [14]. For example, the legume sugar transport protein gene *STP13* and its derivative protein products present in many plants, including legumes, have been found to contribute to basal resistance against both biotrophic and necrotrophic pathogens, which is exacerbated by the specific mutation of one of its amino acids. Hence, the knockout of this gene to modify the amino acid through CRISPR/Cas9 would eliminate its function and prevent or slow pathogen infection in pea [289]. Similarly, the use of transgenic canola lines expressing the pea defence gene (*DRR*206) was able to control *R. solani* infection [138]. This further reiterates the importance of transgenes in disease control. *Agrobacterium tumefaciens*-mediated gene transformation (T-DNA) techniques have led to the introduction of useful genes from wild relatives and unrelated sources into cultivated lines. These have been used to transform pea roots to induce defence-related mechanisms in response to colonization by a model non-pathogenic *Fusarium oxysporum* (*Fo*47) [290]. This T-DNA mediated technique could be applied to transform pea donor parents with disease-resistance traits, and the transgene can be used to create elite inbred lines to broaden genetic pools. Although some of these novel approaches are still in the discovery phase, they are expected to facilitate pea breeding in the near future and improve the accuracy of disease-resistance evaluations. Decisively, the availability of the genome sequence of pea is improving the application of high-throughput genotyping and genetic tools, thus enhancing association mapping studies to allow breeders to gain better insight into pea genetics to select important traits and their use in MAS [18,291]. This high-resolution pea genome could also serve as a model to strengthen the study of pea-phylogeny-related species.

## 5. Concluding Remarks

Soilborne pathogens are difficult to manage, and available control methods are not cost-effective nor environmentally friendly. Considerable efforts have been made to improve pea resistance against soilborne pathogens with some levels of incomplete resistance accumulated in cultivars. However, available sources of resistance are limited, and screening methods are time-consuming, reinforcing the need to improve phenotyping accuracy at an affordable cost by adopting novel technologies. This should be complemented with molecular techniques, although these techniques still require accurate phenotypic data to enhance their results. It is crucial to improve the current phenotyping bottlenecks to complement the available sophisticated genomic technologies for pea rhizospheric disease management. The current advances in powerful high-throughput phenotyping platforms would be useful for verifying genetic data. Correspondingly, the recent accompanying cost reduction in precision molecular genetic tools for genome sequencing and marker-trait discoveries are strengths for future pea resistance breeding. There is a great deal of available information and genetic resources as assets for enhancing pea breeding efforts. Remarkably, the optimised pea reference genome released in 2019 is positively impacting pea breeding strategies at the molecular level. This is expected to foster more precise breeding and plant performance indices to heighten complex trait discovery for genetic gain. Furthermore, different breeding programmes can collaborate to share variable breeding materials and enable the gene pyramiding of useful traits and implement different hybridization regimes with recurrent selections to stabilize acquired resistance genes.

At the same time, there is the need to complement these genetic gains with integrated disease management techniques to sustain and prevent such resistance from deterioration. It is paramount to continue to scout for soil amendment disease suppressers to improve soilborne disease breeding efforts. Management efforts that seek to prevent the establishment of soilborne diseases are the ideal control strategies. Thus, disease surveillance through quarantine, prohibition of planting materials from infested areas, seed treatment and approved seed sources, farmer and technician training and education, laboratory diagnostics, and collaboration among institutions and scientists for quick response to disease outbreaks is necessary. The way forward for the complex nature of rhizospheric diseases calls for more robust multidisciplinary approaches that employ all aspects of biological and socio-scientific understanding and policy directives to enhance environmental sustainability and food security.

## References

[1] Smýkal P., Aubert G., Burstin J., Coyne C.J., Ellis N.T., Flavell A.J., Ford R., Hýbl M., Macas J., Neumann P. (2012). Pea (*Pisum sativum* L.) in the genomic era. Agronomy.

[2] Rubiales D., Ambrose M.J., Domoney C., Burstin J., Pérez-de-la-Vega M., Torres A.M., Cubero J.I., Kole C. (2011). Pea. Genetics, Genomics and Breeding of Cool Season Grain Legumes.

[3] Trněný O., Brus J., Hradilová I., Rathore A., Das R.R., Kopecký P., Coyne C.J., Reeves P., Richards C., Smýkal P. (2018). Molecular evidence for two domestication events in the pea crop. Genes.

[4] Pandey A.K., Rubiales D., Wang Y., Fang P., Sun T., Liu N., Xu P. (2021). Omics resources and omics-enabled approaches for achieving high productivity and improved quality in pea (*Pisum sativum* L.). Theor. Appl. Genet..

[5] Coyne C.J., Kumar S., von Wettberg E.J.B., Marques E., Berger J.D., Redden R.J., Ellis T.N., Brus J., Zablatzká L., Smýkal P. (2020). Potential and limits of exploitation of crop wild relatives for pea, lentil, and chickpea improvement. Legum. Sci..

[6] Pratap A., Das A., Kumar S., Gupta S. (2021). Current Perspectives on Introgression Breeding in Food Legumes. Front. Plant Sci..

[7] Cobos M.J., Satovic Z., Rubiales D., Fondevilla S. (2018). *Er3* gene, conferring resistance to powdery mildew in pea, is located in pea LGIV. Euphytica.

[8] Rubiales D., Fondevilla S., Fernández-Aparicio M. (2021). Development of pea breeding lines with resistance to *Orobanche crenata* derived from pea landraces and wild *Pisum* spp.. Agronomy.

[9] Rubiales D., Osuna-Caballero S., González-Bernal M.J., Cobos M.J., Flores F. (2021). Pea Breeding Lines Adapted to Autumn Sowings in Broomrape Prone Mediterranean Environments. Agronomy.

[10] Das A., Parihar A.K., Saxena D., Singh D., Singha K.D., Kushwaha K.P.S., Chand R., Bal R.S., Chandra S., Gupta S. (2019). Deciphering genotype-by- Environment interaction for targeting test environments and rust resistant genotypes in field pea (*Pisum sativum* L.). Front. Plant Sci..

[11] Varshney R.K., Barmukh R., Roorkiwal M., Qi Y., Kholova J., Tuberosa R., Reynolds M.P., Tardieu F., Siddique K.H. (2021). Breeding custom-designed crops for improved drought adaptation. Adv. Genet..

[12] Rubiales D., González-Bernal M.J., Warkentin T., Bueckert R., Vaz Patto M.C., McPhee K., McGee R., Smýkal P., Hochmuth G. (2019). Advances in pea breeding. Achieving Sustainable Cultivation of Vegetables.

[13] FAOSTAT, Statistical Database. Food and Agriculture Organization of the United Nations. http://www.fao.org/faostat.

[14] Hickey L.T., Hafeez A.N., Robinson H., Jackson S.A., Leal-Bertioli S.C.M., Tester M., Gao C., Godwin I.D., Hayes B.J., Wulff B.B. (2019). Breeding crops to feed 10 billion. Nat. Biotechnol..

[15] Varshney R.K., Bohra A., Roorkiwal M., Barmukh R., Cowling W.A., Chitikineni A., Lam H.M., Hickey L.T., Croser J.S., Bayer P.E. (2021). Fast-forward breeding for a food-secure world. Trends Genet..

[16] Smýkal P., Varshney R.K., Singh V.K., Coyne C.J., Domoney C., Kejnovský E., Warkentin T. (2016). From Mendel’s discovery on pea to today’s plant genetics and breeding: Commemorating the 150th anniversary of the reading of Mendel’s discovery. Theor. Appl. Genet..

[17] Graham P.H., Vance C.P. (2003). Update on Legume Utilization Legumes: Importance and Constraints to Greater Use. Plant Physiol..

[18] Kreplak J., Madoui M.A., Cápal P., Novák P., Labadie K., Aubert G., Gali K.K. (2019). A reference genome for pea provides insight into legume genome evolution. Nat. Genet..

[19] Zander P., Amjath-Babu T.S., Preissel S., Reckling M., Bues A., Schläfke N., Kuhlman T., Bachinger J., Uthes S., Stoddard F. (2016). Grain legume decline and potential recovery in European agriculture: A review. Agron. Sustain. Dev..

[20] Ojiewo C., Monyo E., Desmae H., Boukar O., Mukankusi-Mugisha C., Thudi M., Pandey M.K., Saxena R.K., Gaur P.M., Chaturvedi S.K. (2019). Genomics, genetics and breeding of tropical legumes for better livelihoods of smallholder farmers. Plant Breed..

[21] Döring T.F., Rosslenbroich D., Giese C., Athmann M., Watson C., Vágó I., Kátai J., Tállai M., Bruns C. (2020). Disease suppressive soils vary in resilience to stress. Appl. Soil Ecol..

[22] Vishwakarma K., Kumar N., Shandilya C., Mohapatra S., Bhayana S., Varma A. (2020). Revisiting Plant–Microbe Interactions and Microbial Consortia Application for Enhancing Sustainable Agriculture: A Review. Front. Microbiol..

[23] Müller D.B., Vogel C., Bai Y., Vorholt J.A. (2016). The plant microbiota: Systems-level insights and perspectives. Annu. Rev. Genet..

[24] Latati M., Aouiche A., Tellah S., Laribi A., Benlahrech S., Kaci G., Ouarem F., Ounane S.M. (2017). Intercropping maize and common bean enhances microbial carbon and nitrogen availability in low phosphorus soil under Mediterranean conditions. Eur. J. Soil Biol..

[25] Hamel C., Gan Y.T., Sokolski S., Bainard L.D. (2018). High frequency cropping of pulses modifies soil nitrogen level and the rhizosphere bacterial microbiome in 4-year rotation systems of the semiarid prairie. Appl. Soil Ecol..

[26] Wille L., Messmer M.M., Studer B., Hohmann P. (2019). Insights to plant–microbe interactions provide opportunities to improve resistance breeding against root diseases in grain legumes. Plant Cell Environ..

[27] Chaudhari D., Rangappa K., Das A., Layek J., Basavaraj S., Kandpal B.K., Shouche Y., Rahi P. (2020). Pea (*Pisum sativum* L.) plant shapes its rhizosphere microbiome for nutrient uptake and stress amelioration in acidic soils of the North-East region of India. Front. Microbiol..

[28] Dolgikh E.A., Kusakin P.G., Kitaeva A.B., Tsyganova A.V., Kirienko A.N., Leppyanen I.V., Dolgikh A.V., Ilina E.L., Demchenko K.N., Tikhonovich I.A. (2020). Mutational analysis indicates that abnormalities in rhizobial infection and subsequent plant cell and bacteroid differentiation in pea (*Pisum sativum*) nodules coincide with abnormal cytokinin responses and localization. Ann. Bot..

[29] Rodriguez-Carres M., White G., Tsuchiya D., Taga M., VanEtten H.D. (2008). The supernumerary chromosome of *Nectria haematococca* that carries pea-pathogenicity-related genes also carries a trait for pea rhizosphere competitiveness. Appl. Environ. Microbiol..

[30] Belimov A.A., Shaposhnikov A.I., Syrova D.S., Kichko A.A., Guro P.V., Yuzikhin O.S., Azarova T.S., Sazanova A.L., Sekste E.A., Litvinskiy V.A. (2020). The role of symbiotic microorganisms, nutrient uptake and rhizosphere bacterial community in response of pea (*Pisum sativum* L.) genotypes to elevated Al concentrations in soil. Plants.

[31] Bani M., Cimmino A., Evidente A., Rubiales D., Rispail N. (2018). Pisatin involvement in the variation of inhibition of *Fusarium oxysporum* f. sp. *pisi* spore germination by root exudates of *Pisum* spp. germplasm. Plant Pathol..

[32] Oyserman B.O., Flores S.S., Griffioen T., Pan X., van der Wijk E., Pronk L., Lokhorst W., Nurfikari A., Paulson J.N., Movassagh M. (2022). Disentangling the genetic basis of rhizosphere microbiome assembly in tomato. Nat. Commun..

[33] Whipps J.M. (2001). Microbial interactions and biocontrol in the rhizosphere. J. Exp. Bot..

[34] Williamson-Benavides B.A., Sharpe R.M., Nelson G., Bodah E.T., Porter L.D., Dhingra A. (2021). Identification of Root Rot Resistance QTLs in Pea Using *Fusarium solani* f. sp. *pisi*-Responsive Differentially Expressed Genes. Front. Genet..

[35] Fernández-Aparicio M., García-Garrido J.M., Ocampo J.A., Rubiales D. (2010). Colonisation of field pea roots by arbuscular mycorrhizal fungi reduces *Orobanche* and *Phelipanche* species seed germination. Weed Res..

[36] Harkes P., Van Steenbrugge J.J.M., Van Den Elsen S.J.J., Suleiman A.K.A., De Haan J.J., Holterman M.H.M., Helder J. (2020). Shifts in the active rhizobiome paralleling low *Meloidogyne chitwoodi* densities in fields under prolonged organic soil management. Front. Plant Sci..

[37] Hawes M., Allen C., Turgeon B.G., Curlango-Rivera G., Minh Tran T., Huskey D.A., Xiong Z. (2016). Root border cells and their role in plant defense. Annu. Rev. Phytopathol..

[38] Camborde L., Kiselev A., Pel M.J.C., Le Ru A., Jauneau A., Pouzet C., Dumas B., Gaulin E. (2021). An oomycete effector targets a plant RNA helicase involved in root development and defense. New Phytol..

[39] Wille L., Messmer M.M., Bodenhausen N., Studer B., Hohmann P. (2020). Heritable Variation in Pea for Resistance Against a Root Rot Complex and Its Characterization by Amplicon Sequencing. Front. Plant Sci..

[40] Tosi M., Mitter E.K., Gaiero J., Dunfield K. (2020). It takes three to tango: The importance of microbes, host plant, and soil management to elucidate manipulation strategies for the plant microbiome. Can. J. Microbiol..

[41] Dubey S., Sharma S. (2021). Rhizospheric Engineering by Plant-Mediated Indirect Selection of Microbiome for Agricultural Sustainability. Crit. Rev. Plant Sci..

[42] Hartmann A., Rothballer M., Schmid M. (2008). Lorenz Hiltner, a pioneer in rhizosphere microbial ecology and soil bacteriology research. Plant Soil.

[43] Lucas M.R., Huynh B.L., da Silva Vinholes P., Cisse N., Drabo I., Ehlers J.D., Roberts P.A., Close T.J. (2013). Association studies and legume synteny reveal haplotypes determining seed size in *Vigna unguiculata*. Front. Plant Sci..

[44] Niks R.E., Parlevliet J.E., Lindhout P., Bai Y. (2019). Breeding Crops with Resistance to Diseases and Pests.

[45] Rubiales D., Sivasankar S., Bergvinson D., Gaur P., Kumar S., Beebe S., Tamò M. (2018). Developing pest- and disease-resistant cultivars of grain legumes. Achieving Sustainable Cultivation of Grain Legumes. Volume 1: Advances in Breeding and Cultivation Techniques.

[46] Raaijmakers J.M., Paulitz T.C., Steinberg C., Alabouvette C., Moënne-Loccoz Y. (2009). The rhizosphere: A playground and battlefield for soilborne pathogens and beneficial microorganisms. Plant Soil.

[47] O’Keeffe K.R., Carbone I., Jones C.D., Mitchell C.E. (2017). Plastic potential: How the phenotypes and adaptations of pathogens are influenced by microbial interactions within plants. Curr. Opin. Plant Biol..

[48] Lyu D., Msimbira L.A., Nazari M., Antar M., Pagé A., Shah A., Monjezi N., Zajonc J., Tanney C.A., Backer R. (2021). The coevolution of plants and microbes underpins sustainable agriculture. Microorganisms.

[49] Infantino A., Kharrat M., Riccioni L., Coyne C.J., McPhee K.E., Grünwald N.J. (2006). Screening techniques and sources of resistance to root diseases in cool season food legumes. Euphytica.

[50] Foster K., You M.P., Nietschke B., Edwards N., Barbetti M.J. (2017). Soilborne root disease pathogen complexes drive widespread decline of subterranean clover pastures across diverse climatic zones. Crop Pasture Sci..

[51] Rubiales D., Fondevilla S., Chen W., Gentzbittel L., Higgins T.J.V., Castillejo M.A., Singh K.B., Rispail N. (2015). Achievements and Challenges in Legume Breeding for Pest and Disease Resistance. CRC. Crit. Rev. Plant Sci..

[52] Zitnick-Anderson K., del Río Mendoza L.E., Forster S., Pasche J.S. (2020). Associations among the communities of soil-borne pathogens, soil edaphic properties and disease incidence in the field pea root rot complex. Plant Soil.

[53] Di Pietro A., Madrid M.P., Caracuel Z., Delgado-Jarana J., Roncero M.I.G. (2003). *Fusarium oxysporum*: Exploring the molecular arsenal of a vascular wilt fungus. Mol. Plant Pathol..

[54] Leslie J.F., Summerell B.A. (2006). The Fusarium Laboratory Manual.

[55] Bani M., Rubiales D., Rispail N. (2012). A detailed evaluation method to identify sources of quantitative resistance to *Fusarium oxysporum* f. sp. *pisi* race 2 within a *Pisum* spp. germplasm collection.. Plant Pathol..

[56] Willsey T., Patey J., Vucurevich C., Chatterton S., Carcamo H. (2021). Evaluation of foliar and seed treatments for integrated management of root rot and pea leaf weevil in field pea and faba bean. Crop Prot..

[57] Sampaio A.M., De Sousa Araújo S., Rubiales D., Patto M.C.V. (2020). Fusarium wilt management in legume crops. Agronomy.

[58] Kraft J.M. (1994). Fusarium wilt of peas (a review). Agronomie.

[59] Mcphee K.E., Tullu A., Kraft J.M., Muehlbauer F.J. (1999). Resistance to Fusarium Wilt Race 2 in the *Pisum* Core Collection. J. Am. Soc. Hortic. Sci..

[60] Bani M., Pérez-de-Luque A., Rubiales D., Rispail N. (2018). Physical and chemical barriers in root tissues contribute to quantitative resistance to *Fusarium oxysporum* f. sp. *pisi* in Pea.. Front. Plant Sci..

[61] Kraft J.M., Pfleger F.L. (2001). Compendium of Pea Diseases and Pests.

[62] Shubha K., Dhar S., Choudhary H., Dubey S.C., Sharma R.K. (2016). Identification of resistant sources and inheritance of Fusarium wilt resistance in garden pea (*Pisum sativum* ssp. *hortense*). Indian J. Hortic..

[63] Harveson R.M., Pasche J.S., Porter L., Chen W., Burrows M. (2021). Compendium of Pea Diseases and Pests.

[64] Leslie J.F., Anderson L.L., Bowden R.L., Lee Y.W. (2007). Inter- and intra-specific genetic variation in *Fusarium*. Int. J. Food Microbiol..

[65] Coyne C.J., Porter L.D., Boutet G., Ma Y., McGee R.J., Lesné A., Baranger A., Pilet-Nayel M.L. (2019). Confirmation of Fusarium root rot resistance QTL Fsp-Ps 2.1 of pea under controlled conditions. BMC Plant Biol..

[66] Jha U.C., Bohra A., Pandey S., Parida S.K. (2020). Breeding, genetics, and genomics approaches for improving Fusarium wilt resistance in major grain legumes. Front. Genet..

[67] Rispail N., Bani M., Rubiales D. (2015). Resistance reaction of *Medicago truncatula* genotypes to *Fusarium oxysporum*: Effect of plant age, substrate and inoculation method. Crop Pasture Sci..

[68] Sampaio A.M., Vitale S., Turrà D., Di Pietro A., Rubiales D., van Eeuwijk F., Vaz Patto M.C. (2021). A diversity of resistance sources to *Fusarium oxysporum* f. sp. *pisi* found within grass pea germplasm. Plant Soil..

[69] Leslie J.F., Summerell B.A. (2006). Fusarium laboratory workshops--A recent history. Mycotoxin Res..

[70] Wang R., Huang J., Liang A., Wang Y., Mur L.A.J., Wang M., Guo S. (2020). Zinc and copper enhance cucumber tolerance to fusaric acid by mediating its distribution and toxicity and modifying the antioxidant system. Int. J. Mol. Sci..

[71] Wang M., Liu W., Yan J., Sun P., Chen F., Jiang B., Xie D., Lin Y., Peng Q., He X. (2021). A Transcriptomic Analysis of Gene Expression in Chieh-Qua in Response to Fusaric Acid Stress. Horticulturae.

[72] Kraft J.M., Papavizas G.C. (1983). Use of host resistance, Trichoderma, and fungicides to control soilborne diseases and increase seed yields of peas. Plant Dis..

[73] Riaz R., Khan A., Khan W.J., Jabeen Z., Yasmin H., Naz R., Nosheen A., Hassan M.N. (2021). Vegetable associated *Bacillus* spp. suppress the pea (*Pisum sativum* L.) root rot caused by *Fusarium solani*. Biol. Control.

[74] Summerell B.A., Leslie J.F. (2011). Fifty years of Fusarium: How could nine species have ever been enough?. Fungal Divers..

[75] Saremi H., Okhovvat S.M., Ashrafi S.J. (2011). Fusarium diseases as the main soil borne fungal pathogen on plants and their control management with soil solarization in Iran. Afr. J. Biotechnol..

[76] Dita M., Barquero M., Heck D., Mizubuti E.S.G., Staver C.P. (2018). Fusarium wilt of banana: Current knowledge on epidemiology and research needs toward sustainable disease management. Front. Plant Sci..

[77] Grajal-Martìn M.J., Muehlbauer F.J. (2002). Genomic location of the Fw gene for resistance to Fusarium wilt race 1 in peas. J. Hered..

[78] McClendon M.T., Inglis D.A., McPhee K.E., Coyne C.J. (2002). DNA markers linked to fusarium wilt race 1 resistance in pea. J. Am. Soc. Hortic. Sci..

[79] Okubara P.A., Keller K.E., McClendon M.T., Inglis D.A., McPhee K.E., Coyne C.J. (2015). Y15_999 Fw, a dominant SCAR marker linked to the Fusarium wilt race 1 (Fw) resistance gene in pea. Pisum Genet..

[80] Wohor Z.O., Rispail N., Rubiales D. Evaluation of a *Pisum* spp. Germplasm Collection for the Resistance to *Fusarium oxysporum* f. sp. *pisi* Schlecht and *Orobanche crenata* Forsk. Proceedings of the 9th International Conference on Legume Genetics and Genomics ICLGG.

[81] McPhee K.E., Inglis D.A., Gundersen B., Coyne C.J. (2012). Mapping QTL for Fusarium wilt Race 2 partial resistance in pea (*Pisum sativum*). Plant Breed..

[82] Cheng P., Holdsworth W., Ma Y., Coyne C.J., Mazourek M., Grusak M.A., Fuchs S., McGee R.J. (2015). Association mapping of agronomic and quality traits in USDA pea single-plant collection. Mol. Breed..

[83] Sampaio A.M., Alves M.L., Pereira P., Valiollahi E., Santos C., Šatović Z., Rubiales D., Araújo S.D.S., van Eeuwijk F., Vaz Patto M.C. (2021). Grass pea natural variation reveals oligogenic resistance to *Fusarium oxysporum* f. sp. *pisi*. Plant Genome.

[84] Xue A.G. (2003). Biological control of pathogens causing root rot complex in field pea using *Clonostachys rosea* strain ACM941. Phytopathology.

[85] Smith S.N. (2007). An Overview of Ecological and Habitat Aspects in the Genus *Fusarium* with Special Emphasis on the Soil- Borne Pathogenic Forms. Plant Pathol..

[86] Zitnick-Anderson K., Simons K., Pasche J.S. (2018). Detection and qPCR quantification of seven *Fusarium* species associated with the root rot complex in field pea. Can. J. Plant Pathol..

[87] Tu J.C. (1992). Management of root rot diseases of peas, beans, and tomatoes. Can. J. Plant Pathol..

[88] Sivachandra Kumar N.T., Cox L., Armstrong-Cho C., Banniza S. (2020). Optimization of zoospore production and inoculum concentration of *Aphanomyces euteiches* for resistance screening of pea and lentil. Can. J. Plant Pathol..

[89] Hossain S., Bergkvist G., Berglund K., Mårtensson A., Persson P. (2012). Aphanomyces pea root rot disease and control with special reference to impact of Brassicaceae cover crops. Acta Agric. Scand. Sect. B Soil Plant Sci..

[90] Wu L., Chang K.F., Conner R.L., Strelkov S., Fredua-Agyeman R., Hwang S.F., Feindel D. (2018). *Aphanomyces euteiches*: A Threat to Canadian Field Pea Production. Engineering.

[91] Billard E., Quillévéré-Hamard A., Lavaud C., Pilet-Nayel M.L., Le May C. (2019). Testing of life history traits of a soilborne pathogen in vitro: Do characteristics of oospores change according the strains of *Aphanomyces euteiches* and the host plant infected by the pathogen?. J. Phytopathol..

[92] Gaulin E., Madoui M.A., Bottin A., Jacquet C., Mathé C., Couloux A., Wincker P., Dumas B. (2008). Transcriptome of *Aphanomyces euteiches*: New Oomycete putative pathogenicity factors and metabolic pathways. PLoS ONE.

[93] Pilet-Nayel M.L., Muehlbauer F.J., McGee R.J., Kraft J.M., Baranger A., Coyne C.J. (2005). Consistent quantitative trait loci in pea for partial resistance to *Aphanomyces euteiches* isolates from the United States and France. Phytopathology.

[94] Sharma A., Rani M., Lata H., Thakur A., Sharma P., Kumar P., Jayswal D.K., Rana R.S. (2022). Global dimension of root rot complex in garden pea: Current status and breeding prospective. Crop Prot..

[95] Godebo A.T., Germida J.J., Walley F.L. (2020). Isolation, identification, and assessment of soil bacteria as biocontrol agents of pea root rot caused by *Aphanomyces euteiches*. Can. J. Soil Sci..

[96] Godebo A.T., Wee N.M.J., Yost C.K., Walley F.L., Germida J.J. (2021). A Meta-Analysis to Determine the State of Biological Control of Aphanomyces Root Rot. Front. Mol. Biosci..

[97] Lagerlöf J., Ayuke F., Heyman F., Meijer J. (2020). Effects of biocontrol bacteria and earthworms on *Aphanomyces euteiches* root-rot and growth of peas (*Pisum sativum*) studied in a pot experiment. Acta Agric. Scand. Sect. B–Soil Plant Sci..

[98] Hossain S., Bergkvist G., Glinwood R., Berglund K., Mårtensson A., Hallin S., Persson P. (2015). Brassicaceae cover crops reduce Aphanomyces pea root rot without suppressing genetic potential of microbial nitrogen cycling. Plant Soil.

[99] McGee R.J., Coyne C.J., Pilet-Nayel M.L., Moussart A., Tivoli B., Baranger A., Hamon C., Vandemark G., McPhee K. (2012). Registration of pea germplasm lines partially resistant to *Aphanomyces* root rot for breeding fresh or freezer pea and dry pea types. J. Plant Regist..

[100] Pilet-Nayel M.L., Muehlbauer F.J., McGee R.J., Kraft J.M., Baranger A., Coyne C.J. (2002). Quantitative trait loci for partial resistance to Aphanomyces root rot in pea. Theor. Appl. Genet..

[101] Hamon C., Coyne C.J., McGee R.J., Lesné A., Esnault R., Mangin P., Hervé M., Le Goff I., Deniot G., Roux-Duparque M. (2013). QTL meta-analysis provides a comprehensive view of loci controlling partial resistance to *Aphanomyces euteiches* in four sources of resistance in pea. BMC Plant Biol..

[102] Lavaud C., Lesné A., Piriou C., Le Roy G., Boutet G., Moussart A., Poncet C., Delourme R., Baranger A., Pilet-Nayel M.L. (2015). Validation of QTL for resistance to *Aphanomyces euteiches* in different pea genetic backgrounds using near-isogenic lines. Theor. Appl. Genet..

[103] Desgroux A., L’Anthoëne V., Roux-Duparque M., Rivière J.P., Aubert G., Tayeh N., Moussart A., Mangin P., Vetel P., Piriou C. (2016). Genome-wide association mapping of partial resistance to *Aphanomyces euteiches* in pea. BMC Genom..

[104] Desgroux A., Baudais V.N., Aubert V., Le Roy G., de Larambergue H., Miteul H., Aubert G., Boutet G., Duc G., Baranger A. (2018). Comparative genome-wide-association mapping identifies common loci controlling root system architecture and resistance to *Aphanomyces euteiches* in pea. Front. Plant Sci..

[105] Bonhomme M., Fariello M.I., Navier H., Hajri A., Badis Y., Miteul H., Samac D.A., Dumas B., Baranger A., Jacquet C. (2019). A local score approach improves GWAS resolution and detects minor QTL: Application to *Medicago truncatula* quantitative disease resistance to multiple *Aphanomyces euteiches* isolates. Heredity.

[106] Wu L., Fredua-Agyeman R., Hwang S.F., Chang K.F., Conner R.L., McLaren D.L., Strelkov S.E. (2021). Mapping QTL associated with partial resistance to Aphanomyces root rot in pea (*Pisum sativum* L.) using a 13.2 K SNP array and SSR markers. Theor. Appl. Genet..

[107] Hadwiger L.A. (2008). Pea-Fusarium solani interactions contributions of a system toward understanding disease resistance. Phytopathology.

[108] Baćanović-Šišić J., Šišić A., Schmidt J.H., Finckh M.R. (2018). Identification and characterization of pathogens associated with root rot of winter peas grown under organic management in Germany. Eur. J. Plant Pathol..

[109] Fernandez M.R., Huber D., Basnyat P., Zentner R.P. (2008). Impact of agronomic practices on populations of *Fusarium* and other fungi in cereal and noncereal crop residues on the Canadian Prairies. Soil Tillage Res..

[110] Bodah E.T., Porter L.D., Chaves B., Dhingra A. (2016). Evaluation of pea accessions and commercial cultivars for fusarium root rot resistance. Euphytica.

[111] Singh B.P., Singh G., Krishna K., Nayak S.C., Srinivasa N. (2020). Management of Fungal Pathogens in Pulses: Current Status and Future Challenges.

[112] Mazzola M. (2002). Mechanisms of natural soil suppressiveness to soilborne diseases. Antonie Van Leeuwenhoek Int. J. Gen. Mol. Microbiol..

[113] Jha P.K., Jalali B.L. (2006). Biocontrol of pea root rot incited by *Fusarium solani* f. sp. *pisi* with rhizosphere mycoflora. Indian Phytopathol..

[114] Panth M., Hassler S.C., Baysal-Gurel F. (2020). Methods for Management of Soilborne Diseases in Crop Production. Agriculture.

[115] Grünwald N.J., Coffman V.A., Kraft J.M. (2003). Sources of Partial Resistance to Fusarium Root Rot in the *Pisum* Core Collection. Plant Dis..

[116] Li W.J., Feng J., Chang K.F., Conner R.L., Hwang S.F., Strelkov S.E., Gossen B.D., McLaren D.L. (2012). Microsatellite DNA markers indicate quantitative trait loci controlling resistance to pea root rot caused by *Fusarium avenaceum* (Corda ex Fries) Sacc. Plant Pathol. J..

[117] Feng J., Hwang R., Chang K.F., Conner R.L., Hwang S.F., Strelkov S.E., Gossen B.D., McLaren D.L., Xue A.G. (2011). Identification of microsatellite markers linked to quantitative trait loci controlling resistance to Fusarium root rot in field pea. Can. J. Plant Sci..

[118] Williamson-Benavides B.A., Sharpe R.M., Nelson G., Bodah E.T., Porter L.D., Dhingra A. (2020). Identification of *Fusarium solani* f. sp. *pisi* (*Fsp*) Responsive Genes in *Pisum sativum*. Front. Genet..

[119] Grünwald N.J., Chen W., Larsen R.C. (2004). Pea Diseases and their Management. Diseases of Fruits and Vegetables.

[120] Nel W.J., Duong T.A., Wingfield B.D., Wingfield M.J., de Beer Z.W. (2018). A new genus and species for the globally important, multihost root pathogen *Thielaviopsis basicola*. Plant Pathol..

[121] Abawi G.S., Widmer T.L. (2000). Impact of soil health management practices on soilborne pathogens, nematodes and root diseases of vegetable crops. Appl. Soil Ecol..

[122] Hood M.E., Shew H.D. (1996). Pathogenesis of Thielaviopsis basicola on a susceptible and a resistant cultivar of burley tobacco. Phytopathology.

[123] Niu C., Lister H.E., Nguyen B., Wheeler T.A., Wright R.J. (2008). Resistance to *Thielaviopsis basicola* in the cultivated a genome cotton. Theor. Appl. Genet..

[124] Keijer J. (1996). The initial steps of the infection process in *Rhizoctonia solani*. Rhizoctonia Species: Taxonomy, Molecular Biology, Ecology, Pathology and Disease Control.

[125] Sharma-Poudyal D., Paulitz T.C., Porter L.D., Sharma-Poudyal D. (2015). Characterization and pathogenicity of Rhizoctonia and Rhizoctonia-like spp. From pea crops in the Columbia Basin of Oregon and Washington. Plant Dis..

[126] Beniwal S.P.S., Ahmed S., Gorfu D. (1992). Wilt/root rot diseases of chickpea in Ethiopia. Trop. Pest Manag..

[127] Ketta H., Elkhateeb N., Saleh M., Kamel S. (2021). Efficiency Assessment of Combinations Between *Rhizobium leguminosarum* and *Trichoderma* spp. for Controlling of Pea (*Pisum sativum* L.) Damping-off Disease. Egypt. J. Phytopathol..

[128] Uwaremwe C., Yue L., Liu Y., Tian Y., Zhao X., Wang Y., Xie Z., Zhang Y., Cui Z., Wang R. (2021). Molecular identification and pathogenicity of *Fusarium* and *Alternaria* species associated with root rot disease of wolfberry in Gansu and Ningxia provinces, China. Plant Pathol..

[129] Wille L., Kurmann M., Messmer M.M., Studer B., Hohmann P. (2021). Untangling the Pea Root Rot Complex Reveals Microbial Markers for Plant Health. Front. Plant Sci..

[130] Flower K.C., Hüberli D., Collins S.J., Thomas G., Ward P.R., Cordingley N. (2019). Progression of plant-parasitic nematodes and foliar and root diseases under no-tillage with different crop rotations. Soil Tillage Res..

[131] El_Komy M.H., Hassouna M.G., Abou-Taleb E.M., Al-Sarar A.S., Abobakr Y. (2020). A mixture of *Azotobacter*, *Azospirillum*, and *Klebsiella* strains improves root-rot disease complex management and promotes growth in sunflowers in calcareous soil. Eur. J. Plant Pathol..

[132] Khan M.R., Ashraf S., Rasool F., Salati K.M., Mohiddin F.A., Haque Z. (2014). Field performance of Trichoderma species against wilt disease complex of chickpea caused by *Fusarium oxysporum* f. sp. *ciceri* and *Rhizoctonia solani*. Turk. J. Agric. For..

[133] Hashem A.H., Abdelaziz A.M., Askar A.A., Fouda H.M., Khalil A.M.A., Abd-Elsalam K.A., Khaleil M.M. (2021). Bacillus megaterium-mediated synthesis of selenium nanoparticles and their antifungal activity against *Rhizoctonia solani* in faba bean plants. J. Fungi.

[134] Cubeta M.A., Thomas E., Dean R.A., Jabaji S., Neate S.M., Tavantzis S., Toda T., Vilgalys R., Bharathan N., Fedorova-Abrams N. (2014). Draft genome sequence of the plant-pathogenic soil fungus *Rhizoctonia solani* anastomosis group 3 strain Rhs1AP. Genome Announc..

[135] Zhong J., Chen C.Y., Gao B.D. (2015). Genome sequence of a novel mycovirus of Rhizoctonia solani, a plant pathogenic fungus. Virus Genes.

[136] Chen Y., Su J.E., Qin X.Y., Fan Z.Y., Zhang X.H., Yu Q., Xia Z.Y., Zou C.M., Zhao G.K., Lin Z.L. (2020). A novel putative betapartitivirus isolated from the plant-pathogenic fungus *Rhizoctonia solani*. Arch. Virol..

[137] Akhter W., Bhuiyan M.K.A., Sultana F., Hossain M.M. (2015). Integrated effect of microbial antagonist, organic amendment and fungicide in controlling seedling mortality (*Rhizoctonia solani*) and improving yield in pea (*Pisum sativum* L.). Comptes Rendus Biol..

[138] Wang Y., Fristensky B. (2001). Transgenic canola lines expressing pea defense gene DRR206 have resistance to aggressive blackleg isolates and to *Rhizoctonia solani*. Mol. Breed..

[139] Dölfors F., Holmquist L., Dixelius C., Tzelepis G. (2019). A LysM effector protein from the basidiomycete *Rhizoctonia solani* contributes to virulence through suppression of chitin-triggered immunity. Mol. Genet. Genom..

[140] Liu Y., Hassan S., Kidd B.N., Garg G., Mathesius U., Singh K.B., Anderson J.P. (2017). Ethylene signaling is important for isoflavonoid-mediated resistance to *Rhizoctonia solani* in roots of *Medicago truncatula*. Mol. Plant-Microbe Interact..

[141] Schroeder K.L., Martin F.N., de Cock A.W.A.M., Lévesque C.A., Spies C.F.J., Okubara P.A., Paulitz T.C. (2013). Molecular detection and quantification of pythium species: Evolving taxonomy, new tools, and challenges. Plant Dis..

[142] Kageyama K. (2014). Molecular taxonomy and its application to ecological studies of *Pythium* species. J. Gen. Plant Pathol..

[143] Khalil S.A.M., Nehal S.-M., Nadia G.-G., Mokhtar M.-K. (2020). Field approaches of chemical inducers and bioagents for controlling root diseases incidence of pea (*Pisum sativum* L.) under field conditions. Plant Pathol. J..

[144] Wu W., Ogawa F., Ochiai M., Yamada K., Fukui H. (2020). Common strategies to control pythium disease. Rev. Agric. Sci..

[145] Kraft J.M., Haware M.P., Jiménez-Díaz R.M., Bayaa B., Harrabi M. (1993). Screening techniques and sources of resistance to root rots and wilts in cool season food legumes. Euphytica.

[146] Alcala A.V.C., Paulitz T.C., Schroeder K.L., Porter L.D., Derie M.L., du Toit L.J. (2016). Pythium species associated with damping-off of pea in certified organic fields in the Columbia Basin of central Washington. Plant Dis..

[147] Klepadlo M., Balk C.S., Vuong T.D., Dorrance A.E., Nguyen H.T. (2019). Molecular characterization of genomic regions for resistance to *Pythium ultimum* var. *ultimum* in the soybean cultivar Magellan. Theor. Appl. Genet..

[148] Urrea K., Rupe J., Chen P., Rothrock C.S. (2017). Characterization of seed rot resistance to *Pythium aphanidermatum* in soybean. Crop Sci..

[149] Lin F., Wani S.H., Collins P.J., Wen Z., Li W., Zhang N., McCoy A.G., Bi Y., Tan R., Zhang S. (2020). QTL mapping and GWAS for identification of loci conferring partial resistance to *Pythium sylvaticum* in soybean (*Glycine max* (L.) Merr). Mol. Breed..

[150] Navarro F., Sass M.E., Nienhuis J. (2008). Identification and confirmation of quantitative trait loci for root rot resistance in snap bean. Crop Sci..

[151] Arora H., Sharma A., Sharma S., Haron F.F., Gafur A., Sayyed R.Z., Datta R. (2021). Pythium damping-off and root rot of *Capsicum annuum* L.: Impacts, diagnosis, and management. Microorganisms.

[152] Ai G., Yang K., Ye W., Tian Y., Du Y., Zhu H., Li T., Xia Q., Shen D., Peng H. (2020). Prediction and characterization of RXLR effectors in Pythium species. Mol. Plant-Microbe Interact..

[153] Trudgill D.L., Blok V.C. (2001). Apomictic, Polyphagous Root-Knot Nematodes: Exceptionally Successful and Damaging Biotrophic Root Pathogens. Annu. Rev. Phytopathol..

[154] Mesa-Valle C.M., Garrido-Cardenas J.A., Cebrian-Carmona J., Talavera M., Manzano-Agugliaro F. (2020). Global research on plant nematodes. Agronomy.

[155] Castillo P., Navas-Cortés J.A., Landa B.B., Jiménez-Díaz R.M., Vovlas N. (2008). Plant-parasitic nematodes attacking chickpea and their in planta interactions with rhizobia and phytopathogenic fungi. Plant Dis..

[156] Jones J.T., Haegeman A., Danchin E.G.J., Gaur H.S., Helder J., Jones M.G.K., Kikuchi T., Manzanilla-López R., Palomares-Rius J.E., Wesemael W.M.L. (2013). Top 10 plant-parasitic nematodes in molecular plant pathology. Mol. Plant Pathol..

[157] Dobosz R., Krawczyk R. (2019). Meloidogyne hapla development on growing legume plants–Short Communication. Plant Prot. Sci..

[158] Singh S.K., Hodda M., Ash G.J. (2013). Plant-parasitic nematodes of potential phytosanitary importance, their main hosts and reported yield losses. EPPO Bull..

[159] Kimpinski J., Sturz A.V. (2003). Managing crop root zone ecosystems for prevention of harmful and encouragement of beneficial nematodes. Soil Tillage Res..

[160] Zhang S., Cui S., McLaughlin N.B., Liu P., Hu N., Liang W., Wu D., Liang A. (2019). Tillage effects outweigh seasonal effects on soil nematode community structure. Soil Tillage Res..

[161] Zhang Y., Li S., Li H., Wang R., Zhang K.Q., Xu J. (2020). Fungi–nematode interactions: Diversity, ecology, and biocontrol prospects in agriculture. J. Fungi.

[162] Li X., Liu C., Zhao H., Gao F., Ji G., Hu F., Li H. (2018). Similar positive effects of beneficial bacteria, nematodes and earthworms on soil quality and productivity. Appl. Soil Ecol..

[163] Di Vito M., Greco N. (1994). Control of food legume nematodes in the Mediterranean Basin 1. EPPO Bull..

[164] Sillero J.C., Villegas-Fernández A.M., Thomas J., Rojas-Molina M.M., Emeran A.A., Fernández-Aparicio M., Rubiales D. (2010). Faba bean breeding for disease resistance. Field Crops Res..

[165] Kosterin O.E. (2016). Prospects of the use of wild relatives for pea breeding. Russ. J. Genet. Appl. Res..

[166] Zwart R.S., Thudi M., Channale S., Manchikatla P.K., Varshney R.K., Thompson J.P. (2019). Resistance to Plant-Parasitic Nematodes in Chickpea: Current Status and Future Perspectives. Front. Plant Sci..

[167] Vovlas A., Santoro S., Radicci V., Leonetti P., Castillo P., Palomares-Rius J.E. (2017). Host-suitability of black medick (*Medicago lupulina* L.) and additional molecular markers for identification of the pea cyst nematode Heterodera goettingiana. Eur. J. Plant Pathol..

[168] Jones M.G.K. (1981). Host cell responses to endoparasitic nematode attack: Structure and function of giant cells and syncytia. Ann. Appl. Biol..

[169] Munawar M., Yevtushenko D.P., Castillo P. (2021). Integrative taxonomy, distribution, and host associations of *Geocenamus brevidens* and *Quinisulcius capitatus* from southern Alberta, Canada. J. Nematol..

[170] Whitehead A.G., Bromilow R.H., Tite D.J., Finch P.H., Fraser J.E., French E.M. (1979). Incorporation of granular nematicides in soil to control pea cyst-nematode, *Heterodera goettingiana*. Ann. Appl. Biol..

[171] Green C.D., Williamson K., Dennis E.B., McBurney T. (1981). The effect of oxamyl on the growth of peas attacked by pea cyst nematode. Ann. Appl. Biol..

[172] Dopierata U., Giebel J. (2002). Herbicides can influence the level of pea infestation by *Heterodera goettingiana*. J. Plant Prot..

[173] Di Vito M., Perrino P. (1978). Reaction of Pisum spp. to the attacks of *Heterodera goettingiana*. Nematol. Mediterr..

[174] Bleve-Zacheo T., Melillo M.T., Zacheo G. (1990). Syncytia development in germplasm pea accessions infected with *Heterodera goettingiana*. Nematol Mediterr..

[175] Veronico P., Melillo M.T., Saponaro C., Leonetti P., Picardi E., Jones J.T. (2011). A polygalacturonase-inhibiting protein with a role in pea defence against the cyst nematode *Heterodera goettingiana*. Mol. Plant Pathol..

[176] Veronico P., Giannino D., Melillo M.T., Leone A., Reyes A., Kennedy M.W., Bleve-Zacheo T. (2006). A novel lipoxygenase in pea roots. Its function in wounding and biotic stress. Plant Physiol..

[177] Sharma S.B., Sikora R.A., Greco N., Di Vito M., Caubel G. (1993). Screening techniques and sources of resistance to nematodes in cool season food legumes. Euphytica.

[178] Castillo P., Navas-Cortés J.A., Gomar-Tinoco D., Di Vito M., Jiménez-Díaz R.M. (2003). Interactions between *Meloidogyne artiellia*, the Cereal and Legume Root-Knot Nematode, and *Fusarium oxysporum* f. sp. *ciceris* Race 5 in Chickpea. Phytopathology.

[179] Sturz A.V., Christie B.R. (2003). Beneficial microbial allelopathies in the root zone: The management of soil quality and plant disease with rhizobacteria. Soil Tillage Res..

[180] Haque Z., Khan M.R. (2021). Identification of multi-facial microbial isolates from the rice rhizosphere and their biocontrol activity against *Rhizoctonia solani* AG1-IA. Biol. Control.

[181] Pandey R., Kalra A., Tandon S., Mehrotra N., Singh H.N., Kumar S. (2000). Essential oils as potent source of nematicidal compounds. J. Phytopathol..

[182] Sharma A., Haseeb A., Abuzar S. (2006). Screening of field pea (*Pisum sativum*) selections for their reactions to root-knot nematode (*Meloidogyne incognita*). J. Zhejiang Univ. Sci. B..

[183] Gautam N.K., Marla S.S., Mirza N., Khan Z., Singh B., Wankhede D.P., Gawade B.H. (2017). Evaluation of field pea accessions for root-knot nematode resistance and possible role of NADP dependent malic enzyme gene in host resistance. Indian J. Genet. Plant Breed..

[184] Youssef M., El-Nagdi W. (2019). Differential responses of certain field pea and cowpea cultivars to root-knot nematode, *Meloidogyne incognita* for commercial release. Bull. Natl. Res. Cent..

[185] Hewezi T., Baum T.J. (2013). Manipulation of plant cells by cyst and root-knot nematode effectors. Mol. Plant-Microbe Interact..

[186] Clément M., Ketelaar T., Rodiuc N., Banora M.Y., Smertenko A., Engler G., Abad P., Hussey P.J., de Almeida Engler J. (2009). Actin-depolymerizing factor2-mediated actin dynamics are essential for root-knot nematode infection of Arabidopsis. Plant Cell.

[187] Abad P., Gouzy J., Aury J.M., Castagnone-Sereno P., Danchin E.G., Deleury E., Perfus-Barbeoch L., Anthouard V., Artiguenave F., Blok V.C. (2008). Genome sequence of the metazoan plant-parasitic nematode Meloidogyne incognita. Nat. Biotechnol..

[188] Upadhaya A., Yan G., Pasche J. (2019). Reproduction Ability and Growth Effect of Pin Nematode, *Paratylenchus nanus*, With Selected Field Pea Cultivars. Plant Dis..

[189] Reen R.A., Mumford M.H., Thompson J.P. (2019). Novel Sources of Resistance to Root-Lesion Nematode (*Pratylenchus thornei*) in a New Collection of Wild *Cicer* Species (*C. reticulatum* and *C. echinospermum*) to Improve Resistance in Cultivated Chickpea (*C. arietinum*). Phytopathology.

[190] Kandel S.L., Smiley R.W., Garland-Campbell K., Elling A.A., Huggins D., Paulitz T.C. (2018). Spatial distribution of root lesion nematodes (*Pratylenchus* spp.) in a long-term no-till cropping system and their relationship with soil and landscape properties. Eur. J. Plant Pathol..

[191] Smiley R. (2021). Root-lesion Nematodes Affecting Dryland Cereals in the Semiarid Pacific Northwest USA. Plant Dis..

[192] Taylor S.P., Hollaway G.J., Hunt C.H. (2000). Effect of field crops on population densities of *Pratylenchus neglectus* and *P. thornei* in Southeastern Australia; Part 1: P. neglectus. J. Nematol..

[193] Thompson J.P. (1990). Treatments to eliminate root-lesion nematode (*Pratylenchus thornei* Sher & Allen) from a vertisol. Nematologica.

[194] Taylor S.P., Vanstone V.A., Ware A.H., McKay A.C., Szot D., Russ M.H. (1999). Measuring yield loss in cereals caused by root lesion nematodes (*Pratylenchus neglectus* and *P. thornei*) with and without nematicide. Aust. J. Agric. Res..

[195] Thompson J.P., Reen R.A., Clewett T.G., Sheedy J.G., Kelly A.M., Gogel B.J., Knights E.J. (2011). Hybridisation of Australian chickpea cultivars with wild *Cicer* spp. increases resistance to root-lesion nematodes (*Pratylenchus thornei* and *P. neglectus*). Australas. Plant Pathol..

[196] Rubiales D., Fernández-Aparicio M., Harveson R.M. (2021). Parasitic weed: Broomrape. Compendium of Pea Diseases and Pests.

[197] Rubiales D., Fernández-Aparicio M. (2012). Innovations in parasitic weeds management in legume crops. A review. Agron. Sustain. Dev..

[198] Fernández-Aparicio M., Flores F., Rubiales D. (2016). The effect of *Orobanche crenata* infection severity in faba bean, field pea, and grass pea productivity. Front. Plant Sci..

[199] Fernández-Aparicio M., Yoneyama K., Rubiales D. (2011). The role of strigolactones in host specificity of *Orobanche* and *Phelipanche* seed germination. Seed Sci. Res..

[200] Rubiales D., Moreno M.T., Sillero J.C. (2005). Search for resistance to crenate broomrape (*Orobanche crenata* Forsk.) in pea germplasm. Genet. Resour. Crop Evol..

[201] Rispail N., Dita M.A., González-Verdejo C., Pérez-de-Luque A., Castillejo M.A., Prats E., Román B., Jorrín J., Rubiales D. (2007). Plant resistance to parasitic plants: Molecular approaches to an old foe. New Phytol..

[202] Pérez-de-Luque A., Moreno M.T., Rubiales D. (2008). Host plant resistance against broomrapes (*Orobanche* spp.): Defence reactions and mechanisms of resistance. Ann. Appl. Biol..

[203] Fernández-Aparicio M., Rubiales D., Gregory P. (2021). Advances in understanding plant root response to weedy root parasites. Improving Crop Root Function.

[204] Rubiales D., Fernández-Aparicio M., Wegmann K., Joel D.M. (2009). Revisiting strategies for reducing the seedbank of *Orobanche* and *Phelipanche* spp.. Weed Res..

[205] Fernández-Aparicio M., Sillero J.C., Rubiales D. (2007). Intercropping with cereals reduces infection by *Orobanche crenata* in legumes. Crop Prot..

[206] Fernández-Aparicio M., Emeran A.A., Rubiales D. (2008). Control of Orobanche crenata in legumes intercropped with fenugreek (*Trigonella foenum-graecum*). Crop Prot..

[207] Rubiales D. (2003). Parasitic plants, wild relatives and the nature of resistance. New Phytol..

[208] Mabrouk Y., Zourgui L., Sifi B., Delavault P., Simier P., Belhadj O. (2007). Some compatible *Rhizobium leguminosarum* strains in peas decrease infections when parasitised by *Orobanche crenata*. Weed Res..

[209] Mabrouk Y., Mejri S., Belhadj O. (2016). Biochemical mechanisms of induced resistance by rhizobial lipopolysaccharide in pea against crenate broomrape. Rev. Bras. Bot..

[210] Louarn J., Carbonne F., Delavault P., Bécard G., Rochange S. (2012). Reduced Germination of *Orobanche cumana* Seeds in the Presence of Arbuscular Mycorrhizal Fungi or Their Exudates. PLoS ONE.

[211] Rubiales D., Fernández-Aparicio M., Pérez-de-Luque A., Castillejo M.A., Prats E., Sillero J.C., Rispail N., Fondevilla S. (2009). Breeding approaches for crenate broomrape (*Orobanche crenata* Forsk.) management in pea (*Pisum sativum* L.). Pest Manag. Sci..

[212] Pérez-de-Luque A., Jorrín J., Cubero J.I., Rubiales D. (2005). *Orobanche crenata* resistance and avoidance in pea (*Pisum* spp.) operate at different developmental stages of the parasite. Weed Res..

[213] Rubiales D. (2020). Broomrape threat to agriculture. Outlooks Pest Manag..

[214] Valderrama M.R., Román B., Satovic Z., Rubiales D., Cubero J.I., Torres A.M. (2004). Locating quantitative trait loci associated with *Orobanche crenata* resistance in pea. Weed Res..

[215] Fondevilla S., Fernández-Aparicio M., Satovic Z., Emeran A.A., Torres A.M., Moreno M.T., Rubiales D. (2010). Identification of quantitative trait loci for specific mechanisms of resistance to *Orobanche crenata* Forsk. in pea (*Pisum sativum* L.). Mol. Breed..

[216] Fondevilla S., Flores F., Emeran A.A., Kharrat M., Rubiales D. (2017). High productivity of dry pea genotypes resistant to crenate broomrape in Mediterranean environments. Agron. Sustain. Dev..

[217] Fernández-Aparicio M., Flores F., Rubiales D. (2009). Recognition of root exudates by seeds of broomrape (*Orobanche* and *Phelipanche*) species. Ann. Bot..

[218] Dita M.A., Die J.V., Román B., Krajinski F., Küster H., Moreno M.T., Cubero J.I., Rubiales D. (2009). Gene expression profiling of *Medicago truncatula* roots in response to the parasitic plant *Orobanche crenata*. Weed Res..

[219] Castillejo M.Á., Maldonado A.M., Dumas-Gaudot E., Fernández-Aparicio M., Susín R., Rubiales D., Jorrín J.V. (2009). Differential expression proteomics to investigate responses and resistance to *Orobanche crenata* in *Medicago truncatula*. BMC Genom..

[220] Castillejo M.Á., Amiour N., Dumas-Gaudot E., Rubiales D., Jorrín J.V. (2004). A proteomic approach to studying plant response to crenate broomrape (*Orobanche crenata*) in pea (*Pisum sativum*). Phytochemistry.

[221] Rubiales D. (2018). Can we breed for durable resistance to broomrapes?. Phytopathol. Mediterr..

[222] Varshney R.K., Kudapa H., Pazhamala L., Chitikineni A., Thudi M., Bohra A., Gaur P.M., Janila P., Fikre A., Kimurto P. (2015). Translational Genomics in Agriculture: Some Examples in Grain Legumes. CRC. Crit. Rev. Plant Sci..

[223] Thudi M., Palakurthi R., Schnable J.C., Chitikineni A., Dreisigacker S., Mace E., Srivastava R.K., Satyavathi C.T., Odeny D., Tiwari V.K. (2021). Genomic resources in plant breeding for sustainable agriculture. J. Plant Physiol..

[224] Tivoli B., Baranger A., Avila C.M., Banniza S., Barbetti M., Chen W., Davidson J., Lindeck K., Kharrat M., Rubiales D. (2006). Screening techniques and sources of resistance to foliar diseases caused by major necrotrophic fungi in grain legumes. Euphytica.

[225] Burud I., Lange G., Lillemo M., Bleken E., Grimstad L., From P.J. (2017). Exploring robots and UAVs as phenotyping tools in plant breeding. IFAC-PapersOnLine.

[226] Chen C.Y., Butts C.L., Dang P.M., Wang M.L. (2015). Advances in phenotyping of functional traits. Phenomics in Crop Plants: Trends, options and Limitations.

[227] Cazzola F., Bermejo C.J., Guindon M.F., Cointry E. (2020). Speed breeding in pea (*Pisum sativum* L.), an efficient and simple system to accelerate breeding programs. Euphytica.

[228] Rubiales D., Pérez-de-Luque A., Fernández-Aparico M., Sillero J.C., Román B., Kharrat M., Khalil S., Joel D.M., Riches C. (2006). Screening techniques and sources of resistance against parasitic weeds in grain legumes. Euphytica.

[229] Furbank R.T., Tester M. (2011). Phenomics–technologies to relieve the phenotyping bottleneck. Trends Plant Sci..

[230] Fernández-Aparicio M., Rubiales D. (2010). Characterisation of resistance to crenate broomrape (*Orobanche crenata* Forsk.) in *Lathyrus cicera* L. Euphytica.

[231] Fernández-Aparicio M., Moral A., Kharrat M., Rubiales D. (2012). Resistance against broomrapes (*Orobanche* and *Phelipanche* spp.) in faba bean (*Vicia faba*) based in low induction of broomrape seed germination. Euphytica.

[232] Marzougui A., Rajendran A., Mattinson D.S., Ma Y., McGee R.J., Garcia-Perez M., Ficklin S.P., Sankaran S. (2022). Evaluation of biogenic markers-based phenotyping for resistance to Aphanomyces root rot in field pea. Inf. Process. Agric..

[233] Divyanth L.G., Marzougui A., Gonzalez-Bernal M.J., McGee R.J., Rubiales D., Sankaran S. (2022). Evaluation of effective class-balancing techniques for CNN-based assessment of Aphanomyces root rot resistance in pea. Sensors.

[234] Araus J.L., Kefauver S.C., Zaman-Allah M., Olsen M.S., Cairns J.E. (2018). Translating high-throughput phenotyping into genetic gain. Trends Plant Sci..

[235] Araus J.L., Cairns J.E. (2014). Field high-throughput phenotyping: The new crop breeding frontier. Trends Plant Sci..

[236] Araus J.L., Kefauver S.C. (2018). Breeding to adapt agriculture to climate change: Affordable phenotyping solutions. Curr. Opin. Plant Biol..

[237] Jung J., Maeda M., Chang A., Bhandari M., Ashapure A., Landivar-Bowles J. (2021). The potential of remote sensing and artificial intelligence as tools to improve the resilience of agriculture production systems. Curr. Opin. Biotechnol..

[238] Quirós Vargas J.J., Zhang C., Smitchger J.A., McGee R.J., Sankaran S. (2019). Phenotyping of plant biomass and performance traits using remote sensing techniques in pea (*Pisum sativum* L.). Sensors.

[239] Yao L., Van De Zedde R., Kowalchuk G. (2021). Recent developments and potential of robotics in plant eco-phenotyping. Emerg. Top. Life Sci..

[240] Nguyen G.N., Norton S.L., Rosewarne G.M., James L.E., Slater A.T. (2018). Automated phenotyping for early vigour of field pea seedlings in controlled environment by colour imaging technology. PLoS ONE.

[241] Rispail N., Rubiales D. (2015). Rapid and efficient estimation of pea resistance to the soil-borne pathogen *Fusarium oxysporum* by infrared imaging. Sensors.

[242] Bohar R., Chitkineni A., Varshney R.K. (2020). Genetic molecular markers to accelerate genetic gains in crops. Biotechniques.

[243] Moreira F.F., Oliveira H.R., Volenec J.J., Rainey K.M., Brito L.F. (2020). Integrating High-Throughput Phenotyping and Statistical Genomic Methods to Genetically Improve Longitudinal Traits in Crops. Front. Plant Sci..

[244] Bates G.H. (1937). A Device for the Observation of Root Growth in the Soil. Nature.

[245] Kuijken R.C.P., van Eeuwijk F.A., Marcelis L.F.M., Bouwmeester H.J. (2015). Root phenotyping: From component trait in the lab to breeding. J. Exp. Bot..

[246] Cabrera J., Conesa C.M., del Pozo J.C. (2022). May the dark be with roots: A perspective on how root illumination may bias in vitro research on plant–environment interactions. New Phytol..

[247] Yee M.O., Kim P., Li Y., Singh A.K., Northen T.R., Chakraborty R. (2021). Specialized Plant Growth Chamber Designs to Study Complex Rhizosphere Interactions. Front. Microbiol..

[248] Jeudy C., Adrian M., Baussard C., Bernard C., Bernaud E., Bourion V., Busset H., Cabrera-Bosquet L., Cointault F., Han S. (2016). RhizoTubes as a new tool for high throughput imaging of plant root development and architecture: Test, comparison with pot grown plants and validation. Plant Methods.

[249] Taylor H.M., Upchurch D.R., McMichael B.L. (1990). Applications and limitations of rhizotrons and minirhizotrons for root studies. Plant Soil.

[250] Klepper B., Kaspar T.C. (1994). Rhizotrons: Their Development and Use in Agricultural Research. Agron. J..

[251] Bodner G., Nakhforoosh A., Arnold T., Leitner D. (2018). Hyperspectral imaging: A novel approach for plant root phenotyping. Plant Methods.

[252] Rahman G., Sohag H., Chowdhury R., Wahid K.A., Dinh A., Arcand M., Vail S. (2020). SoilCam: A fully automated minirhizotron using multispectral imaging for root activity monitoring. Sensors.

[253] Ghosh S., Watson A., Gonzalez-Navarro O.E., Ramirez-Gonzalez R.H., Yanes L., Mendoza-Suárez M., Simmonds J., Wells R., Rayner T., Green P. (2018). Speed breeding in growth chambers and glasshouses for crop breeding and model plant research. Nat. Protoc..

[254] Bhatta M., Sandro P., Smith M.R., Delaney O., Voss-Fels K.P., Gutierrez L., Hickey L.T. (2021). Need for speed: Manipulating plant growth to accelerate breeding cycles. Curr. Opin. Plant Biol..

[255] Rubiales D., Pérez-de-Luque A., Joel D.M., Alcántara C., Sillero J.C. (2003). Characterization of resistance in chickpea to crenate broomrape (*Orobanche crenata*). Weed Sci..

[256] Fernández-Aparicio M., Kisugi T., Xie X., Rubiales D., Yoneyama K. (2014). Low strigolactone root exudation: A novel mechanism of broomrape (*Orobanche* and *Phelipanche* spp.) resistance available for faba bean breeding. J. Agric. Food Chem..

[257] Gurney A.L., Grimanelli D., Kanampiu F., Hoisington D., Scholes J.D., Press M.C. (2003). Novel sources of resistance to *Striga hermonthica* in *Tripsacum dactyloides*, a wild relative of maize. New Phytol..

[258] Gurney A.L., Slate J., Press M.C., Scholes J.D. (2006). A novel form of resistance in rice to the angiosperm parasite *Striga hermonthica*. New Phytol..

[259] Kavuluko J., Kibe M., Sugut I., Kibet W., Masanga J., Mutinda S., Wamalwa M., Magomere T., Odeny D., Runo S. (2021). GWAS provides biological insights into mechanisms of the parasitic plant (Striga) resistance in sorghum. BMC Plant Biol..

[260] Esquinas-Alcázar J. (2005). Protecting crop genetic diversity for food security: Political, ethical and technical challenges. Nat. Rev. Genet..

[261] Bariana H.S., Bansal U.K. (2016). Breeding for Disease Resistance. Encycl. Appl. Plant Sci..

[262] Varshney R.K., Glaszmann J.C., Leung H., Ribaut J.M. (2010). More genomic resources for less-studied crops. Trends Biotechnol..

[263] Varshney R.K., Dubey A. (2009). Novel genomic tools and modern genetic and breeding approaches for crop improvement. J. Plant Biochem. Biotechnol..

[264] Annicchiarico P., Nazzicari N., Pecetti L., Romani M., Ferrari B., Wei Y., Brummer E.C. (2017). GBS-Based Genomic Selection for Pea Grain Yield under Severe Terminal Drought. Plant Genome.

[265] Leonforte A., Sudheesh S., Cogan N.O.I., Salisbury P.A., Nicolas M.E., Materne M., Forster J.W., Kaur S. (2013). SNP marker discovery, linkage map construction and identification of QTLs for enhanced salinity tolerance in field pea (*Pisum sativum* L.). BMC Plant Biol..

[266] Aryamanesh N., Zeng Y., Byrne O., Hardie D.C., Al-Subhi A.M., Khan T., Siddique K.H.M., Yan G. (2014). Identification of genome regions controlling cotyledon, pod wall/seed coat and pod wall resistance to pea weevil through QTL mapping. Theor. Appl. Genet..

[267] Dachapak S., Somta P., Naito K., Tomooka N., Kaga A., Srinives P. (2019). Detection of quantitative trait loci for salt tolerance in zombi pea [*Vigna vexillata* (L.) A. Rich]. Euphytica.

[268] Barilli E., Carrillo-Perdomo E., Cobos M.J., Kilian A., Carling J., Rubiales D. (2020). Identification of potential candidate genes controlling pea aphid tolerance in a *Pisum fulvum* high-density integrated DArTseq SNP-based genetic map. Pest Manag. Sci..

[269] Du X.F., Li Y.B., Han X., Ahmad W., Li Q. (2020). Using high-throughput sequencing quantitatively to investigate soil nematode community composition in a steppe-forest ecotone. Appl. Soil Ecol..

[270] Nasiri J., Haghnazari A., Saba J. (2009). Genetic diversity among varieties and wild species accessions of pea (*Pisum sativum* L.) based on SSR markers. Afr. J. Biotechnol..

[271] Siol M., Jacquin F., Chabert-Martinello M., Smýkal P., Le Paslier M.C., Aubert G., Burstin J. (2017). Patterns of genetic structure and linkage disequilibrium in a large collection of pea germplasm. G3.

[272] Varshney R.K. (2016). Exciting journey of 10 years from genomes to fields and markets: Some success stories of genomics-assisted breeding in chickpea, pigeonpea and groundnut. Plant Sci..

[273] Kankanala P., Nandety R.S., Mysore K.S. (2019). Genomics of Plant Disease Resistance in Legumes. Front. Plant Sci..

[274] Le Signor C., Aimé D., Bordat A., Belghazi M., Labas V., Gouzy J., Young N.D., Prosperi J.M., Leprince O., Thompson R.D. (2017). Genome-wide association studies with proteomics data reveal genes important for synthesis, transport and packaging of globulins in legume seeds. New Phytol..

[275] Zitnick-Anderson K., Oladzadabbasabadi A., Jain S., Modderman C., Osorno J.M., McClean P.E., Pasche J.S. (2020). Sources of Resistance to Fusarium solani and Associated Genomic Regions in Common Bean Diversity Panels. Front. Genet..

[276] Kwon S.J., Smýkal P., Hu J., Wang M., Kim S.J., McGee R.J., McPhee K., Coyne C.J. (2013). User-friendly markers linked to Fusarium wilt race 1 resistance Fw gene for marker-assisted selection in pea. Plant Breed..

[277] Shirasawa K., Sasaki K., Hirakawa H., Isobe S. (2021). Genomic region associated with pod color variation in pea (*Pisum sativum*). G3 Genes Genomes Genet..

[278] Dinglasan E., Periyannan S., Hickey L.T. (2022). Harnessing adult-plant resistance genes to deploy durable disease resistance in crops. Essays Biochem..

[279] Castillejo M.Á., Fernández-Aparicio M., Rubiales D. (2012). Proteomic analysis by two-dimensional differential in gel electrophoresis (2D DIGE) of the early response of *Pisum sativum* to *Orobanche crenata*. J. Exp. Bot..

[280] Castillejo M.Á., Bani M., Rubiales D. (2015). Understanding pea resistance mechanisms in response to *Fusarium oxysporum* through proteomic analysis. Phytochemistry.

[281] Burstin J., Salloignon P., Chabert-Martinello M., Magnin-Robert J.B., Siol M., Jacquin F., Chauveau A., Pont C., Aubert G., Delaitre C. (2015). Genetic diversity and trait genomic prediction in a pea diversity panel. BMC Genom..

[282] Annicchiarico P., Nazzicari N., Laouar M., Thami-Alami I., Romani M., Pecetti L. (2020). Development and proof-of-concept application of genome-enabled selection for pea grain yield under severe terminal drought. Int. J. Mol. Sci..

[283] Bohra A., Pandey M.K., Jha U.C., Singh B., Singh I.P., Datta D., Chaturvedi S.K., Nadarajan N., Varshney R.K. (2014). Genomics-assisted breeding in four major pulse crops of developing countries: Present status and prospects. Theor. Appl. Genet..

[284] Varshney R.K., Tuberosa R. (2013). Translational genomics in crop breeding for biotic stress resistance: An introduction. Transl. Genom. Crop Breed. Vol. I Biot. Stress.

[285] Zargar S.M., Raatz B., Sonah H., Muslima N., Bhat J.A., Dar Z.A., Agrawal G.K., Rakwal R. (2015). Recent advances in molecular marker techniques: Insight into QTL mapping, GWAS and genomic selection in plants. J. Crop Sci. Biotechnol..

[286] Sagan M., Huguet T., Duc G. (1994). Phenotypic characterization and classification of nodulation mutants of pea (*Pisum sativum* L.). Plant Sci..

[287] Dalmais M., Schmidt J., Le Signor C., Moussy F., Burstin J., Savois V., Aubert G., Brunaud V., De Oliveira Y., Guichard C. (2008). UTILLdb, a *Pisum sativum* in silicoforward and reverse genetics tool. Genome Biol..

[288] Tayeh N., Aubert G., Pilet-Nayel M.L., Lejeune-Hénaut I., Warkentin T.D., Burstin J. (2015). Genomic tools in pea breeding programs: Status and perspectives. Front. Plant Sci..

[289] Gupta M., Dubey S., Jain D., Chandran D. (2021). The *Medicago truncatula* sugar transport protein 13 and its Lr67res-like variant confer powdery mildew resistance in legumes via defense modulation. Plant Cell Physiol..

[290] Benhamou N., Garand C. (2001). Cytological analysis of defense-related mechanisms induced in pea root tissues in response to colonization by nonpathogenic *Fusarium oxysporum Fo*47. Phytopathology.

[291] Gali K.K., Tar’an B., Madoui M.A., van der Vossen E., van Oeveren J., Labadie K., Berges H., Bendahmane A., Lachagari R.V., Burstin J. (2019). Development of a sequence-based reference physical map of pea (*Pisum sativum* L.). Front. Plant Sci..

